# Distinct neuroprotective and anti-inflammatory effects of Kampo formulas ninjinyoeito and juzentaihoto in depression-like SAMP8 mice

**DOI:** 10.3389/fphar.2025.1600176

**Published:** 2025-10-24

**Authors:** Akiko Maruko, Naoki Ito, Kenshiro Oshima, Akinori Nishi, Yoshinori Kobayashi, Norihiro Okada

**Affiliations:** ^1^ Department of Pharmacognosy, School of Pharmacy, Kitasato University, Tokyo, Japan; ^2^ Laboratory of Kampo Clinical Research, Oriental Medicine Research Center, School of Pharmacy, Kitasato University, Tokyo, Japan; ^3^ TSUMURA Advanced Technology Research Laboratories, Research & Development Division, TSUMURA & Co., Ibaraki, Japan; ^4^ Oriental Medicine Research Center, School of Pharmacy, Kitasato University, Tokyo, Japan

**Keywords:** Kampo, SAMP8, depression, hippocampus, frailty, juzentaihoto, ninjinyoeito

## Abstract

**Introduction:**

In contemporary aging societies, preventing and ameliorating mental and physical frailty is essential. Kampo formulas, including ninjinyoeito (NYT) and juzentaihoto (JTT), have been used traditionally to treat frailty in the elderly. NYT has been reported to alleviate psychological frailty such as depression and anxiety. This study aimed to clarify the mechanisms underlying the effects of these two Kampo formulas in the early stages of neurodegeneration associated with psychiatric disorders.

**Methods:**

Genes affected by Kampo formulas were comprehensively investigated by administering JTT or NYT to senescence accelerated mouse prone 8 (SAMP8) mice, from 7 weeks, and by RNA sequencing of the hippocampus at 19 weeks when depression and anxiety behaviors typically emerge. Additionally, we examined the impact of these Kampo formulas on neuroinflammation induced by lipopolysaccharide (LPS).

**Results:**

The two Kampo formulas alleviated the depressive-like behavior of SAMP8 mice, as demonstrated by the restoration of microglial cell activation, DNA repair, stress-responsive transcription factor expression, and nervous system development-related gene expression. However, the NYT-administrated group presented a greater number of recovered genes than did the JTT-administrated group, and NYT additionally suggests that the potential inhibition of age-related mitochondrial dysfunction and increased oxidative stress. The administration of LPS resulted in elevated expression levels of immune and inflammation-related genes and increased astrocyte activity in SAMP8 mice. JTT mitigated these effects by suppressing the expression of the LPS receptor TLR4 and its downstream target NF-κB. In contrast to JTT, NYT maintained and increased the expression of genes associated with neuroprotective functions in microglia.

**Discussion:**

The two Kampo formulas exerted neuroprotective effects by enhancing neural and glial stress responses in the early stages of neurodegeneration. Under condition of acute inflammation, JTT and NYT alleviated neuronal damage via the suppression of microglial activity and the enhancement of microglial neuroprotection, respectively. These findings provide novel insights into the mechanism of action of NYT, which has been reported to ameliorate psychological frailty associated with aging, and further suggest that JTT may exert effects against inflammatory neurodegeneration.

## 1 Introduction

Frailty is defined as a state in which muscle strength and mental and physical vitality decline with age and is classified into “physical frailty,” such as sarcopenia and chronic disease; “psychological frailty,” such as depression, anxiety and mild cognitive impairment (MCI); and “social frailty,” such as living alone; however, all three are closely related (Tsutsumimoto et al., 2017; Zhao et al., 2023). Psychological frailty is a complex concept with a multifactorial construct that includes mood problems, cognitive problems, mental health problems, and fatigue-related problems. Although its assessment criteria remain to be clearly defined, the general concept emphasizes vulnerability at the mental or spiritual level, and depression and anxiety are primarily used for its definition (Zhao et al., 2023). Psychological frailty is associated with subsequent memory loss and is known to increase the risk of dementia (Lu et al., 2021; Yin et al., 2024). In addition, a decline in vitality due to psychological frailty can readily result in undernutrition and a loss of muscle mass due to anorexia and weight loss, which are also associated with physical frailty (Auyeung et al., 2011; Sanford, 2017). Consequently, both psychological and physical frailty need to be addressed at an early stage.

Ninjinyoeito (NYT) and juzentaihoto (JTT) are traditional Japanese Kampo formulas that supplement “Ki” (vital energy) and “Ketsu” (blood) and are used to restore vitality to elderly individuals who have lost their energy and physical strength. Both NYT and JTT have the same effects and efficacy and are used to treat constitutional decline from disease, fatigue, anorexia, night swear, cold limbs, and anemia. However, NYT may also be beneficial for patients with psychological frailty. NYT reportedly decreases anxiety and depression scores in frail patients with chronic obstructive pulmonary disease (COPD) (Hirai et al., 2020) and alleviates frailty and anorexia in patients with mild cognitive impairment and Alzheimer’s disease (AD) (Okahara et al., 2023; Ohsawa et al., 2017).

Polygalae Radix [*Polygalaceae*; *Polygala tenuifolia* Willdenow], which is contained in NYT, has been reported in many studies to have antidepressant and neuroprotective effects (Jiang et al., 2021). Citri unshiu Pericarpium [*Rutaceae*; *Citrus unshiu* Markowicz, *Citrus reticulata* Blanco] also has anxiolytic effects (Ito et al., 2013) and improved cognitive function by promoting myelin formation (Sato et al., 2011). Furthermore, Ginseng Radix [*Araliaceae*; *Panax ginseng* C.A. Meyer] -derived some ginsenosides in both NYT and JTT induce anti-inflammatory effects in microglia (Im, 2020) and alleviate depression-like behavior resulting from chronic stress (Guo et al., 2021; Zhang et al., 2021). Rehmanniae Radix [*Scrophulariaceae*; *Rehmannia glutinosa Liboschitz* var. purpurea Makino, *R. glutinosa* Liboschitz] has also been reported to exhibit neuroprotective effects via anti-inflammatory and antioxidant effects (Yang et al., 2020); thus, the clear differences and mechanisms of action between the two Kampo formulas remain unclear.

Senescence-accelerated mouse prone 8 (SAMP8) mice is a line developed from AKR/J mice and exhibit more rapid aging compared with senescence-accelerated mouse resistant 1 (SAMR1), a control group from the same strain that exhibits normal aging (Takeda et al., 1991). A multitude of studies have demonstrated that age-related declines in avoidance adaptation, spatial memory processing, and spatial memory and learning are clearly observed in SAMP8 mice at 4–12 months of age and that age-related AD-like neuropathological and neuromorphological changes are evident from approximately 6 months of age (Cheng et al., 2014). SAMP8 mice also exhibit age-related emotional disorders (Chen et al., 2007; Cheng et al., 2014), and changes in anxiety and depression-like behavior begin to be observed at approximately 4 months of age (Yanai and Endo, 2016; Ito et al., 2020; Ito et al., 2022), making it possible to use SAMP8 mice as a model of aging with psychological frailty preceding cognitive dysfunction.

Although the mechanism of the pathogenesis of depression is not yet clear, it has recently been shown that inflammation and immune disorders are involved as causes of psychiatric disorders, including depression (neuroinflammatory hypothesis) (Patel, 2013; Bullmore, 2018; Woelfer et al., 2019; Okada et al., 2024). In particular, during inflammatory conditions such as infection and stress, microglial activity and microglial senescence have been proposed to play roles in the development of depression (Yirmiya et al., 2015; Ransohoff, 2016; Wang et al., 2022). Consistently, lipopolysaccharide (LPS), a gram-negative bacterial endotoxin that activates microglia, triggers a neuroinflammatory response, causes depression-like symptoms in mice and is used to study the mechanisms of inflammation-related depression (Ito et al., 2022; Yin et al., 2023). Therefore, to test whether the effects of JTT and NYT involve modulation of neuroinflammation, we employed an LPS-induced inflammation model. This model allowed us to specifically examine whether these Kampo formulas exert antidepressant effects through anti-inflammatory mechanisms.

In this study, we investigated how the administration of NYT and JTT affects gene expression levels in the hippocampus of SAMP8 mice, which exhibit depressive-like behavior, in the early stage of aging and to determine the site of action differences during neuroinflammation induced by LPS. Our study revealed that both Kampo formulas alleviated depressive-like behavior but the affected gene clusters ware distinctive, particularly in their differential impact on microglia during acute inflammation.

## 2 Materials and methods

### 2.1 Materials

#### 2.1.1 Ninjinyoeito (NYT) and juzentaihoto (JTT)

The extract powders of NYT (TJ-108, lot no. 392155300) and JTT (TJ-48, lot no. 392150800) were provided as preservative-free pure powder from Tsumura & Co. (Tokyo, Japan). Both formulas were reviewed and evaluated by the Pharmaceuticals and Medical Devices Agency (PMDA) and subsequently approved by the Minister of Health, Labour and Welfare in 1986 (approval no. 16100AMZ03305000 for NYT; 16100AMZ01124000 for JTT).

NYT extract powder 6 g was produced by boiling a mixture of 12 dried botanical drugs, whereas JTT extract powder 5 g was produced by boiling a mixture of 10 dried botanical drugs. All botanical drugs are listed in the Japanese Pharmacopoeia (JP) 18th edition (2021). In both cases, the botanical drugs were boiled, the resulting extract was concentrated, and then spray-dried. The detailed names and compositions of the botanical drugs are summarized in Table 1. The quality of both extracts was standardized according to Good Manufacturing Practices established by the Japanese Ministry of Health, Labor and Welfare. Chemical characterization was performed by Tsumura & Co. using three-dimensional HPLC analysis (Supplementary Image 1 for NYT and Supplementary Image 2 for JTT).

**TABLE 1:** Fomulas of ninjinyoeito (NYT) and juzentaihoto (JTT).

Component botanical drugs	Family name	Species name	Japanese pharmacopoeia (JP) 18th edition	Weight (g)	
				NYT	JTT
Rehmanniae Radix	*Scrophulariaceae*	*Rehmannia glutinosa Liboschitz* var. purpurea Makino, *Rehmannia glutinosa* Liboschitz	Rehmannia Root	4.0	3.0
Angelicae acutilobae Radix	*Umbelliferae*	*Angelicae acutiloba* Kitagawa, *Angelica acutiloba* Kitagawa *var. sugiyamae* Hikino	Japanese Angelica Root	4.0	3.0
Atractylodis Rhizoma	*Compositae*	*Atractylodes japonica* Koidzumi ex Kitamura, *Atractylodes macrocephala* Koidzumi	Atractylodes Rhizome	4.0	_
Poria	*Polyporaceae*	*Wolfiporia cocos* Ryvarden et Gilbertson	Poria Sclerotium	4.0	3.0
Ginseng Radix	*Araliaceae*	*Panax ginseng* C.A. Meyer	Ginseng	3.0	3.0
Cinnamomi Cortex	*Lauraceae*	*Cinnamomum cassia* J. Presl	Cinnamon Bark	2.5	3.0
Polygalae Radix	*Polygalaceae*	*Polygala tenuifolia* Willdenow	Polygala Root	2.0	_
Paeoniae Radix	*Paeoniaceae*	*Paeonia lactiflora* Pallas	Peony Root	2.0	3.0
Citri unshiu Pericarpium	*Rutaceae*	*Citrus unshiu* Markowicz, *Citrus reticulata* Blanco	Citrus Unshiu Peel	2.0	_
Astragali Radix	*Leguminosae*	*Astragalus mongholicus* Bunge, *Astragalus membranaceus* Bunge	Astragalus Root	1.5	3.0
Glycyrrhizae Radix	*Leguminosae*	*Glycyrrhiza uralensis* Fischer, *Glycyrrhiza glabra* Linné	Glycyrrhiza	1.0	1.5
Schisandrae Fructus	*Schisandraceae*	*Schisandra chinensis* Baillon	Schisandra Fruit	1.0	_
Cnidii Rhizoma	*Umbelliferae*	*Cnidium officinale* Makino	Cnidium Rhizome	_	3.0
Atractylodis lanceae Rhizoma	*Compositae*	Atractylodes lancea De Candolle, Atractylodes chinensis Koidzumi	Atractylodes Lancea Rhizome	_	3.0

Both Kampo formulas were standardized and quantified by Tsumura & Co. For JTT, according to the JP, the extract prepared at the specified ratio was confirmed by HPLC to contain not less than 1.8 mg of ginsenoside Rb1, 26–78 mg of paeoniflorin, and 10–30 mg of glycyrrhizic acid per extract. For NYT, glycyrrhizic acid, paeoniflorin, and hesperidin were quantified by HPLC in accordance with the “Determination of Ether-soluble Extract” described in the General Tests of the JP. In addition to the pharmacopoeial standards, these Kampo formulas were subjected to Tsumura’s in-house quality control tests, which are more stringent than the official requirements, ensuring consistent quality.

#### 2.1.2 Experimental diets and dosage calculation of Kampo formulations

NYT and JTT extract powders were mixed with regular chow (FR-2) at Funabashi Farm Co., Ltd. (Chiba, Japan) to prepare diets containing 2.0% NYT and 1.5% JTT, respectively. In our model (average body weight 30 g, daily food intake 3 g), the 1.5% JTT and 2.0% NYT diets provided 1,500 mg/kg/day and 2,000 mg/kg/day, respectively. These doses correspond to approximately 1.5-fold (JTT) and 1.6-fold (NYT) the human dose when calculated using the human equivalent dose (HED) method (Nair and Jacob, 2016), which is within the reported non-toxic range for Kampo extracts.

Calculation method: The clinical daily doses are 5 g JTT extract and 6 g NYT extract for a 60-kg adult, corresponding to 83.3 mg/kg and 100.0 mg/kg in humans, respectively. Using the standard K_m_ factors (for body surface area conversion: human = 37, mouse = 3) (Nair and Jacob, 2016), these values convert to 121.62 mg/kg/day for JTT and 162.16 mg/kg/day for NYT in mice.

$$\text{HED }\left(\text{mg}/\text{kg}\right)=\text{Animal does }\left(\text{mg}/\text{kg}\right)\times \left(\text{Animal }K_{m}/\text{Human }K_{m}\right)$$

$$\text{JTT}:\text{HED}=1,500\text{mg}/\text{kg}/\text{day }x\;\left(3/37\right)=121.62\text{mg}/\text{kg}/\text{day}$$

$$\text{NYT}:\text{HED}=2000\text{mg}/\text{kg}/\text{day }x\;\left(3/37\right)=162.16\text{mg}/\text{kg}/\text{day}$$

These concentrations were also used in previous studies (Yamasaki et al., 2008; Iwasawa et al., 2021).

### 2.2 Animals and general protocol

Male SAMP8 and normal control SAMR1 mice were obtained at 5 weeks of age from Japan SLC (Hamamatsu, Japan) and allowed to acclimate for 1 week after arrival. The mice were housed at a constant temperature (23 °C ± 2 °C) and humidity (55% ± 10%) with a 12-h light/dark cycle, and had access to regular chow (FR-2) and water *ad libitum*. The SAMP8 mice were randomly divided into the following three groups and fed the following diets beginning at 7 weeks of age for 12 weeks: FR-2 containing 2.0% (w/w) NYT, FR-2 containing 1.5% (w/w) JTT, and FR-2. SAMR1 mice were fed FR-2. For RNA-sequencing, 19-week-old mice were intraperitoneally injected with lipopolysaccharide (LPS, 10 mL/kg body weight; serotype O55:B5, 0.33 mg/kg; Sigma, St. Louis, MO, United States) or saline. To clarify the study design and grouping, the experimental conditions are summarized in Table 2. Body weight was measured at 7, 13 and 19 weeks. The mice utilized in the behavioral experiments were housed in the same condition in different groups at different times, but did not receive intraperitoneal administration of saline or LPS. All the animal experiments were approved by the Institutional Animal Care and Use Committee of Kitasato University and were performed in accordance with the Guidelines for the Care and Use of Laboratory Animals of Kitasato University and the National Research Council Guide for the Care and Use of Laboratory Animals in Japan. Every effort was made to minimize the number of animals used and their suffering.

**TABLE 2:** Study design and groups by administration.

Strain	Group	Diet/Formula administration	Age at start	Duration	Administration at 19 weeks	Notes
SAMR1	Control	FR-2	7 weeks	12 weeks	LPS (0.33 mg/kg, i.p.)	N = 5
					Saline, i.p.	N = 5
SAMP8	Control	FR-2	7 weeks	12 weeks	LPS (0.33 mg/kg, i.p.	N = 5
					saline, i.p.	N = 5
SAMP8	NYT group	FR-2 + 2% (w/w) NYT	7 weeks	12 weeks	LPS (0.33 mg/kg, i.p.)	N = 5
					saline, i.p.	N = 5
SAMP8	JTT group	FR-2 + 1.5% (w/w) JTT	7 weeks	12 weeks	LPS (0.33 mg/kg, i.p.)	N = 5
					saline, i.p.	N = 5

### 2.3 Open field test (OFT)

Nineteen-week-old mice were placed in an opaque grey open field box (40 × 40 × 40 cm) and were allowed to explore freely for 5 min under high light condition (120–130 lux). The time spent in the center, middle and periphery zones and the total distance traveled were recorded by a video tracking system (EthoVision 3.0; Noldus, Wageningen, Netherlands). Anxiety-like behaviors were evaluated by the time spent in periphery zones. The OFT was performed over 2 days between 12:00 and 17:00 after acclimation to a habituation room for 1 h.

### 2.4 Tail suspension test (TST)

The immobility time in an inescapable situation served as an assessment of depression-like behavior (Steru et al., 1985; Ito et al., 2020). Mice were suspended 50 cm above the floor by their tails using experimental clips (Yamashitagiken, Tokushima, Japan) attached to a hook connected to a steel bar for 6 min. A mouse was considered immobile only when it ceased struggling and hung motionless. All behaviors were videotaped and the duration of immobility during the last 4 min of the TST was recorded. The TST was conducted between 12:00 and 14:00 after acclimation to a habituation room for 1 h.

### 2.5 Hippocampus and prefrontal cortex collection

Twenty-four hours after LPS or saline injection, the mice were decapitated, and the brain of each mouse was collected. Each brain was frozen in liquid nitrogen and stored at −80 °C until further processing. The brain was sliced into approximately 1-mm thick coronal sections, and the hippocampus (Bregma −2.6 ∼ −3.2 mm) and areas including the prefrontal cortex (PFC) (Bregma 1.4 ∼ 2.0 mm) were excised on dry ice. The hippocampus was immediately immersed in RNA-later solution (Thermo Fisher Scientific, Carlsbad, CA, United States) and stored at 4 °C. The PFC was stored at −80 °C.

### 2.6 RNA extraction and RNA-sequencing (RNA-seq)

RNA extraction was performed with a Pure Link RNA Mini kit (Invitrogen, Carlsbad, CA). Briefly, approximately 20–30 mg of hippocampus was homogenized in 600 μL of buffer, 240 μL of lysis buffer and 360 μL of TRIzol-LS (Thermo Fisher Scientific) using a BioMasher disposable homogenizer (Nippi, Tokyo, Japan). One hundred microliters of 1-Bromo-3-chloropropane (BCP) was added to the homogenate, followed by mixing and centrifugation at 12,000 × g for 15 min at 4 °C. An equal volume of 70% ethanol was added to the aqueous layer, and the resultant RNA was purified using spin columns. The quality of total RNA was checked with a Qubit 4.0 (Thermo Fisher Scientific) and 4200TapeStation system (Agilent Technologies, Santa Clara, CA). Each total RNA sample had an RIN >7, indicating sufficient quality for preparing sequencing libraries. Total RNA was used for RNA-seq. Library preparation and Illumina NovaSeq sequencing were conducted by Azenta Life Sciences (South Plainfield, NJ), with approximately 35 million (150-base pairs, paired-end) reads per library.

### 2.7 Quality check and filtering of RNA-seq data and mapping analysis

To preprocess the sequencing data, cutadapt v.1.16 (Martin, 2011) was used to remove Illumina adapter sequences, followed by the removal of the poly(A) sequence using fastx_clipper software in the fastx toolkit software package v.0.0.14 (https://github.com/agordon/fastx_toolkit). Low-quality bases or sequences were trimmed using fastq_quality_trimmer software (parameters: -t 20 -l 30 -Q 33) and fastq_quality_filter software (parameters: -q 20 -p 80 -Q 33), both of which are included in the fastx toolkit. During this process, any reads missing one of the pairs were removed using Trimmomatic v.0.38 (Bolger et al., 2014). Reads containing mouse rRNA, tRNA, or phiX sequences (Illumina control sequence) were removed using Bowtie 2 v. 2.3.4.1 (Langmead and Salzberg, 2012). A second processing step was performed to remove any unpaired reads using bam2fastq. After these filtering steps were complete, 20 million reads of each forward and reverse sequence per sample were mapped to mouse genome build GRCm38 using TopHat v2.1.1 (Kim et al., 2019). The mouse genome sequence was downloaded from iGenomes of Illumina (http://jp.support.illumina.com/sequencing/sequencing_software/igenome.html). Multiple mapped reads were removed using SAMtools (parameters: SAMtools view -q 4) (Li et al., 2009). Uniquely mapped reads were counted by gene annotation (Ensembl release 81) using featureCounts v.1.6.2 (Liao et al., 2013). The counted values were normalized using the trimmed mean of M (TMM) method with the edgeR (Robinson et al., 2009; Robinson and Oshlack, 2010) library in R software v.4.3.1 and used for expression analysis. Principal component analysis (PCA) plots were generated for genes whose expression levels with TMM values greater than 10 using the ggplot2 library in R.

### 2.8 Analysis of differential expression genes (DEGs)

Genes with significant differential expression (P < 0.05) in two-group comparisons were identified using edgeR. P values for the differential expression of genes were obtained via the likelihood ratio test. DEGs were subjected to Gene Ontology (GO) biological analysis (http://www.geneontology.org/) and the Kyoto Encyclopedia of Genes and Genomes (KEGG) analysis (https://www.genome.jp/kegg/) using DAVID, a functional annotation online database (v.2024q2) (https://david.ncifcrf.gov/). Fisher’s exact test was applied to identify significant GO terms and pathways, and the threshold of significance was defined by the P value and false discovery rate. Gene Set Enrichment Analysis (GSEA) (Mootha et al., 2003; Subramanian et al., 2005) was conducted using log fold-change (logFC) values derived from the comparison of gene expression between the two groups. The analysis was conducted in R software (version 4.3.1) with the clusterProfiler package (version 4.14.6).

### 2.9 Immunoblotting

The PFC was homogenized in RIPA Buffer (50 mM Tris pH 7.4, 150 mM NaCl, 1% NP-40, 0.5% deoxycholic acid sodium monohydrate, 0.1% SDS, 10 mM NaF, and 1 mM EDTA) containing a protease inhibitor cocktail (Wako). The samples were kept on ice for 30 min and centrifuged at 10,000 × g for 10 min at 4 °C. The protein concentrations in the supernatants were measured with Pierce BCA assay Kits in accordance with the manufacturer’s instructions (Thermo Scientific). To prepare samples for gel electrophoresis, the samples were mixed with 4X sample buffer (250 mM Tris pH6.8, 40% Glycerol, 8% SDS, and Bromophenol blue) containing 0.1 M dithiothreitol (Tokyo Chemical Industry. Japan) and boiled for 5 min at 95 °C.

Proteins were separated via 8%, 10% or 12% SDS-PAGE and transferred onto a polyvinylidene fluoride membrane (GE Healthcare, Japan), which was blocked with 5% milk in TBS containing 0.1% Tween-20 and incubated for 1 h at room temperature, with the following primary antibodies: anti-MAP2 (1:5000; rabbit polyclonal, 17490-1-AP, Proteintech, United States), anti-PSD95 (1:1000; rabbit polyclonal, 20665-1-AP, Proteintech), anti-synaptophysin (SNP) (1:20000; rabbit polyclonal, 17785-1-AP, Proteintech), anti-TLR4 (1:2000; rabbit polyclonal, 19811-1-AP, Proteintech), anti-TNF alpha (1:1000; rabbit polyclonal, 17590-1-AP, Proteintech), anti-IL-1 beta (1:3000; rabbit polyclonal, 26048-1-AP, Proteintech), anti-NDUFB8 (1:1000; rabbit monoclonal, F1631, Selleck.co.jp), and anti-GAPDH (1:1000; mouse monoclonal, sc-32233, Santa Cruz). After incubation with a secondary antibody (HRP- conjugated goat anti-rabbit; SA00001-2 or goat anti-mouse; SA00001-1, 1:15000, Proteintech) for 1 h at room temperature, signals were enhanced using ECL Prime Western blotting Detection Reagent (GE Healthcare) and photographed with a LumiCube Plus imaging system (Liponics Inc., Japan). Band intensities were analyzed using NIH ImageJ software (http://imagej.nih.gov/ij/).

### 2.10 Quantitative RT-PCR analysis

To analyze mRNA expression, extracted total RNA was reverse transcribed into complementary DNA. Quantitative reverse transcription PCR (RT-qPCR) was performed on a StepOne Real-Time PCR system (Applied Biosystems) with a Power SYBR™ Green RNA-to-CT 1-Step Kit (Fisher Scientific). The expression of target genes was normalized to that of Gapdh. The results were calculated via the 2^−ΔΔCT^ method. The primer pairs used were as follows: mouse *Gapdh* primer, forward, 5′- TGA​TGG​GTG​TGA​ACC​ACG​AG -3′; reverse, 5′- GCC​CTT​CCA​CAA​TGC​CAA​AG -3′; mouse *Tlr4* primer, forward, 5′- AGA​TCT​GAG​CTT​CAA​CCC​CTT -3′; reverse, 5′-GTC​TCC​ACA​GCC​ACC​AGA​TT -3′; and mouse *Cd14* primer, forward, 5′- GAA​GCA​GAT​CTG​GGG​CAG​TT -3′; reverse, 5′- CGC​AGG​GCT​CCG​AAT​AGA​AT -3′

### 2.11 Periodic acid–schiff (PAS) staining

The brains of mice subjected to behavior tests (19 weeks old) were utilized for periodic acid–Schiff (PAS) staining. Four brains from each group of mice were used. Frozen brains were fixed in 10% formalin, embedded in paraffin, and cut into two 30 µm thick coronal sections. The bregma coordinates of the 2 sections correspond to approximately −1.7 mm to −2.4 mm. The sections were stained with Schiff’s reagent and counterstained with hematoxylin and eosin (HE). These procedures were performed by Genostaff Co., Ltd. (Genostaff, Tokyo, Japan). The analyses of the hippocampal area, the length of the dentate gyrus, and the cell count were conducted using ImageJ software.

### 2.12 Statistical analysis

R statistical software v.4.3.1 was used for the statistical analyses. All the data are expressed as means ± standard deviations (SDs), and the number of samples used (N) is indicated in the figure legends. The distribution of the data was tested via the Shapiro‒ Wilk test. For multiple comparison tests, the Tukey HSD test was used when the data were normally distributed, and Bonferroni correction was used when the data were nonnormally distributed. The data for two groups were analyzed by Student’s t test. Significance levels were defined as p < 0.05 (*), p < 0.01 (**), p < 0.001 (***) and nonsignificant (n.s.).

## 3 Results

### 3.1 Kampo formulas alleviate lethargy and weight loss

**FIGURE 1:**
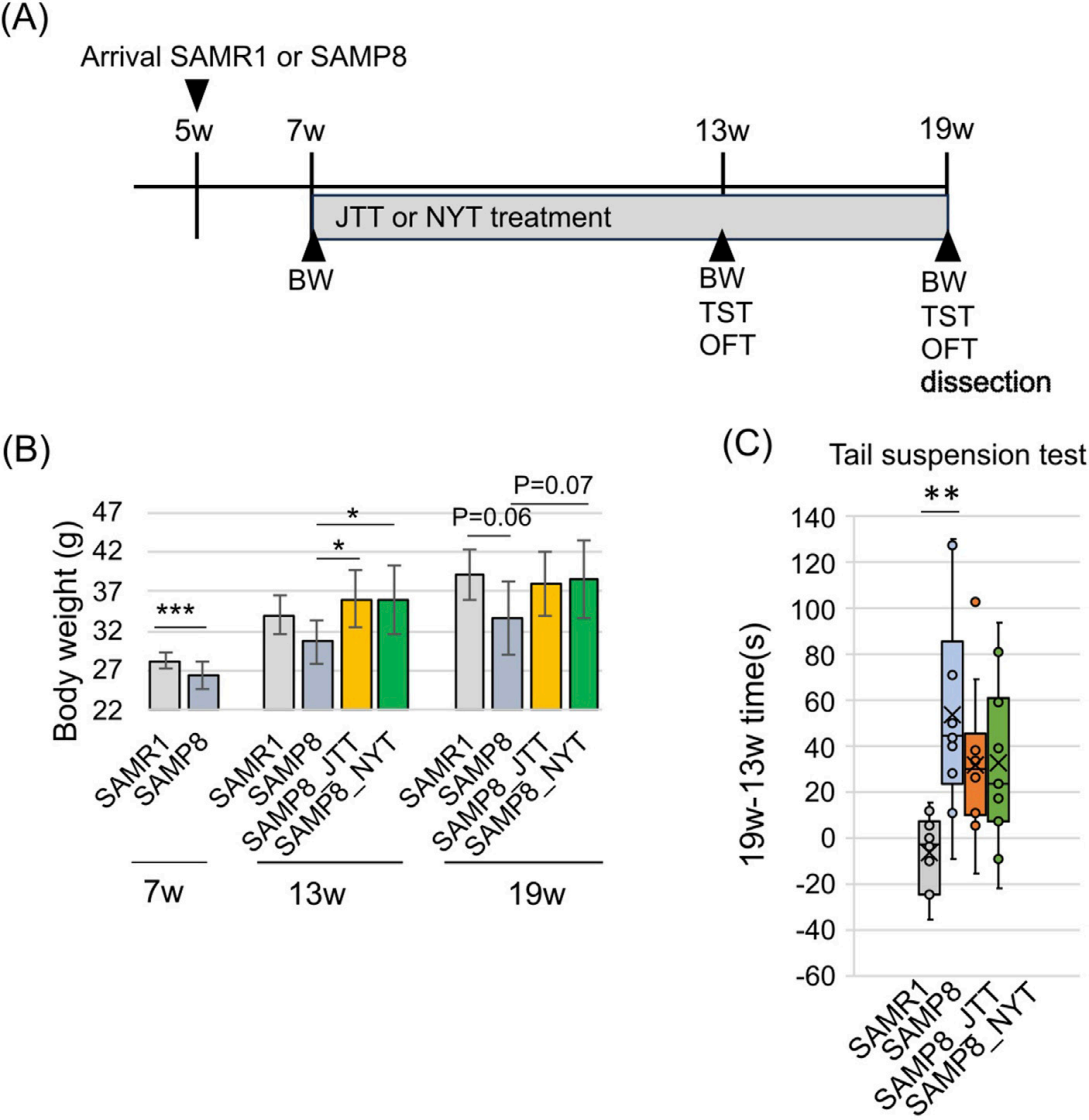
Changes in body weight and behavior with age. **(A)** Schematic representation of the experimental schedule. BW: body weight, TST: tail suspension test, OFT: open field test. **(B)** Change in body weight with Kampo administration. The bars represent means ± SD. *p < 0.05 (Tukey HSD) ***p < 0.001 at 7w (Student’s t-test). **(C)** Variation in immobility time in the TST. The box plot shows the changes in immobility time at 13 and 19 weeks in the same individual. The bottom and top of the box represent the 25th and 75th percentiles, respectively. The crosses represent the means, and bars indicate the maximum and minimum values. **p < 0.01 (Tukey HSD). SAMR1, SAMP8, SAMP8_JTT: n = 10, SAMP8_NYT: n = 11.

We measured body weight and conducted behavioral tests to assess depression-like behavior according to the time schedule shown in Figure 1A. Consistent with reports that SAMP8 mice are leaner than SAMR1 mice beginning at 2 months of age (Pacesova et al., 2022), body weight was significantly lower for SAMP8 mice than SAMR1 mice at 7 weeks but increases with subsequent Kampo-administration to the same level as the observed for SAMR1 mice; body weight was significantly higher for both NYT- and JTT-administrated mice than for SAMP8 mice at 13 weeks (Figure 1B). TSTs were performed to assess depression-like behavior. The data revealed a difference in immobility time between 19 and 13 weeks (Figure 1C); SAMP8 mice were markedly more lethargic than SAMR1 mice were, and JTT or NYT-administration tended to prevent the worsening of lethargy. In the OFT, at 10 weeks, SAMP8 mice spent more time along the periphery and less time in the intermediate zone, indicative of anxiety-like behavior, than SAMR1 mice did (Supplementary Figure S1); however, there was no recovery in Kampo administration. Compared with SAMR1 mice, SAMP8 mice exhibited hyperactivity (Supplementary Figure S1: distance traveled, time traveled, and speed traveled) (Sawano et al., 2013; Tsumagari et al., 2021), potentially indicating age-related motor and emotional dysregulation (Pacesova et al., 2022), effects that were also not altered by Kampo. Consequently, the findings suggest that the lethargic behavior exhibited by SAMP8 mice is not attributable to a decline in spontaneous locomotor activity and that the suppression of lethargic behavior by Kampo formulas is not associated with a decrease in spontaneous locomotor activity. Both JTT and NYT prevented the worsening of age-related weight loss and lethargy in SAMP8 mice.

### 3.2 Changes in gene expression in the hippocampus of SAMP8 mice

**FIGURE 2:**
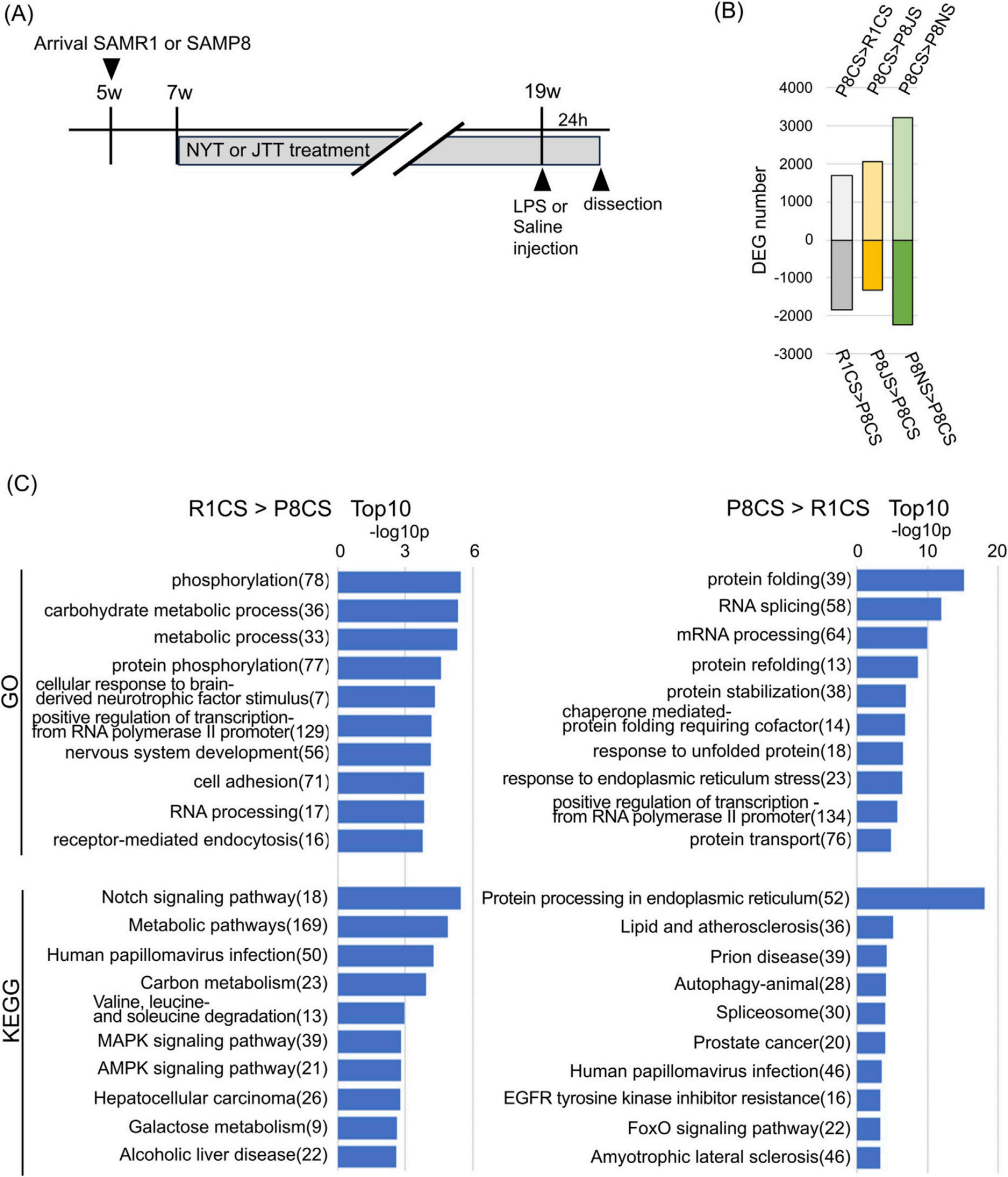
Gene expression and GO analysis of the hippocampus. **(A)** Schematic representation of the experimental schedule. **(B)** Differential gene expression number versus P8CS group (p < 0.05, using the likelihood ratio test). **(C)** Gene ontology analysis of biological processes and KEGG pathway analysis of R1CS > P8CS genes (left) and P8CS > R1CS genes (right). Gene numbers are shown to the right of the terms. Abbreviations: R1CS: SAMR1-control food-saline injection; P8CS: SAMP8-control food-saline injection; P8JS: SAMP8-JTT food-saline injection; P8NS: SAMP8-NYT food-saline injection; LPS: lipopolysaccharide.

RNA-seq was performed to assess changes in hippocampal genes at 19 weeks. LPS was administered intraperitoneally as a single dose to determine the effects of JTT or NYT on acute inflammatory stress, and sampled were collected 24 h later; mice injected with saline served as the control group (Figure 2A). PCA, using only those genes whose expression levels had TMM values greater than 10 (Supplementary Figure S2), revealed that the saline group (dashed line) was clearly separated from the SAMR1 (R1CS), SAMP8 (P8CS), JTT (P8JS), and NYT (P8NS) groups. In the LPS-administrated group (solid line), the SAMP8 (P8CL) and JTT (P8JL) groups overlapped, and their gene expression patterns were similar. PCA analysis with 95% confidence ellipses (stat_ellipse, level = 0.95) showed that all replicates clustered within their respective groups and that no outliers were detected. As shown in Figure 2B, 1701 upregulated and 1831 downregulated DEGs were identified between the P8CS and R1CS groups. Similarly, when the JTT group (P8JS) was compared with the P8CS group, 1309 genes were upregulated (P8JS > P8CS), and 2073 genes were downregulated (P8CS > P8JS) (Figure 2B). When the NYT group was compared with the P8CS group, 2240 genes were upregulated (P8NS > P8CS), and 3210 genes were downregulated (P8CS > P8NS), indicating that many genes were altered by NYT. The list of these DEGs is shown in Supplementary Table S6.

To identify genes altered by premature aging, GO biological process and KEGG pathway analyses were performed for genes whose expression was upregulated or downregulated in P8CS compared to R1CS (Figure 2C). The genes downregulated in P8CS were enriched in metabolic process, protein phosphorylation, nervous system development, cell adhesion, and cell signaling pathways, suggesting decreased energy production, neurogenesis and neural signaling in 19-week-old SAMP8 mice (Figure 2C, left). The genes whose expression was increased in P8CS were enriched in protein folding, RNA splicing, and autophagy-related genes, suggesting the potential for aberrant splicing and an increase in misfolded proteins and their processing in SAMP8 mice (Figure 2C, right).

### 3.3 NYT protects cranial nerves in premature aging

**FIGURE 3:**
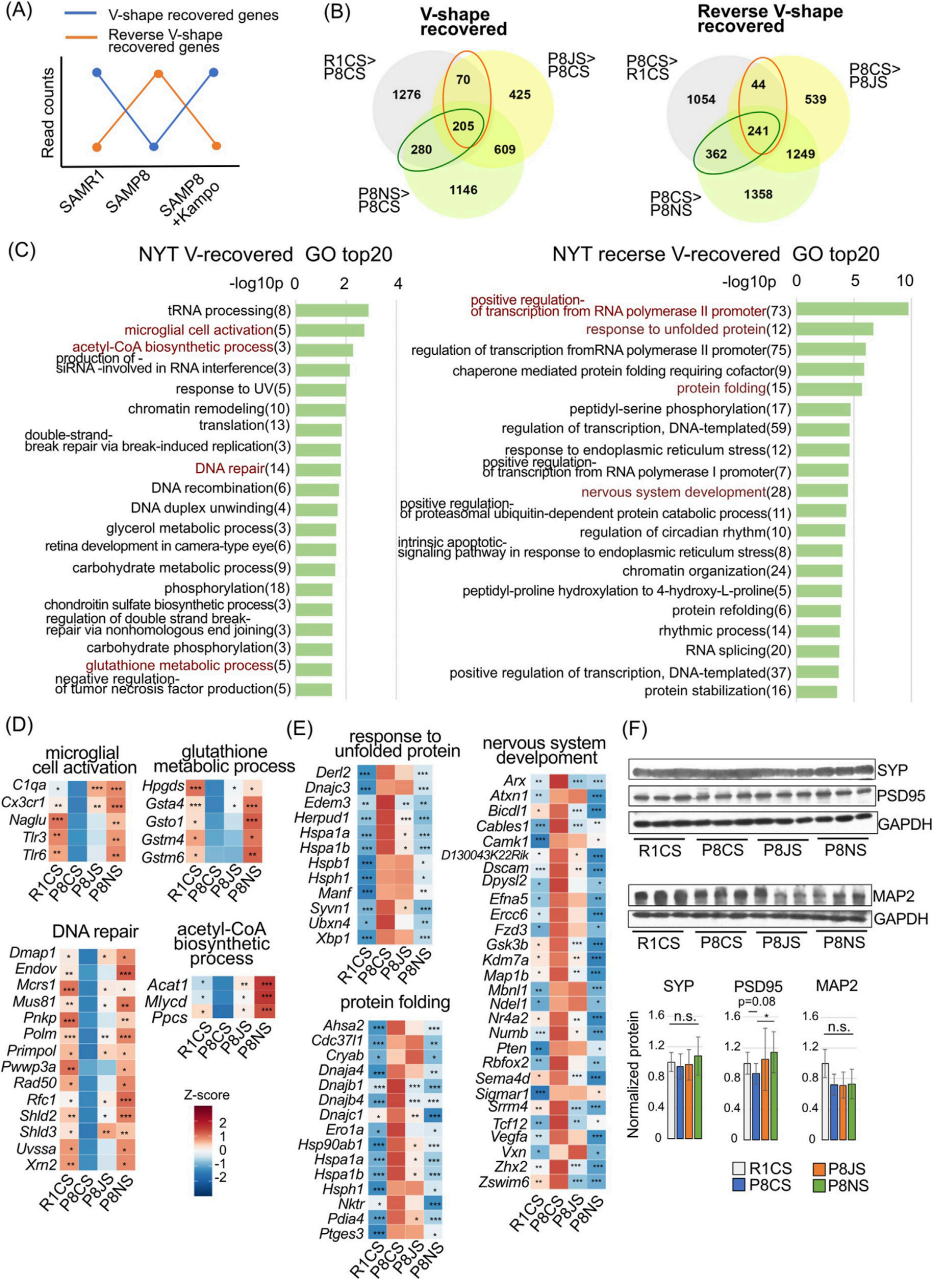
Gene expression and GO analyses of hippocampus. **(A)** Genes whose expression was significantly downregulated in SAMP8 mice compared with SAMR1 mice and whose expression was significantly upregulated by administration of Kampo formulas in SAMP8 mice, i.e., the blue line, are referred to as V-shaped recovered genes. Genes that were significantly upregulated in the SAMP8 mice as compared with the SAMR1 mice and that were significantly downregulated by Kampo formulas, i.e., the orange line, are referred to as reverse V-shaped recovered genes. **(B)** Venn diagram showing the genes differentially expressed between the P8CS group vs. the R1CS, P8JS, or P8NS group. The numbers in circles indicate the number of genes with statistically significant changes in expression (P < 0.05) vs. the P8CS group, as determined via the likelihood ratio test. The orange round frame indicates genes whose expression was restored by JTT, and the green round frame indicates genes whose expression was restored by NYT. **(C)** Gene ontology analysis of the biological processes associated with NYT-V-shaped recovered genes (left) and NYT-reverse V-shaped recovered genes (right). Gene numbers are shown to the right of the term. **(D)** Heatmap of the differentially expressed genes included in the GO terms of the NYT-V-shaped recovered genes in Figure 3C. **(E)** Heatmap of differentially expressed genes included in the GO terms of the NYT-reverse V-shaped recovered genes in Figure 3C. **(F)** Representative images of Western blots of MAP2, SYP and PSD-95 in the prefrontal cortex; the bar graph shows the quantification of relative protein levels. GAPDH was used as a loading control. The data are shown as means ± SDs (n = 5). Experiments were independently repeated 2–3 times. n.s., no significant difference; *p < 0.05 (Bonferroni correction) In **(D,E)**, *P < 0.05, **P < 0.01, and ***P < 0.001 (n = 5) vs. the P8CS group, according to the likelihood ratio test. Abbreviations: R1CS: SAMR1-control food-saline injection; P8CS: SAMP8-control food-saline injection; P8JS: SAMP8-JTT food-saline injection; P8NS: SAMP8-NYT food-saline injection.

To confirm whether the Kampo ameliorated the gene expression alterations observed in SAMP8 mice, we extracted genes whose expression was restored by JTT or NYT. The genes whose expression was downregulated in SAMP8 mice compared with that in SAMR1 mice but whose expression was significantly upregulated by Kampo formulas were named V-shaped recovered genes (Figure 3A: blue line), whereas the genes whose expression was upregulated in SAMP8 mice compared with that in SAMR1 mice but whose expression was significantly downregulated by Kampo formulas were named reverse V-shaped recovered genes (Figure 3A: orange line). As shown in the Venn diagram, the number of recovered genes in the NYT group was greater than that in the JTT group, and approximately 70%–80% of the genes recovered in the JTT group were common to the NYT group (Figure 3B). Therefore, we performed GO analysis of the genes recovered from the NYT group (Figure 3C). In reverse V-shaped recovery, the top significantly enriched biological processes were regulation of transcription, response to unfolded protein, and nervous system development (Figure 3C right). Similar terms were obtained from the GO analysis of recovery genes in the JTT group; however, the number of genes included was smaller than that in NYT group (Supplementary Figure S3A). In reverse V-shaped recovery, genes were enriched in response to unfolded protein or protein folding and included many genes encoding heat shock proteins and cochaperones (HSP family and DNAJ family) (Figure 3E). As heat shock proteins are known to increase with age and stress (Soti and Csermely, 2003), it is hypothesized that the hippocampus of SAMP8 mice contains elevated levels of defective proteins that do not fold properly at 19 weeks. However, this increase in defective proteins may be suppressed by NYT and partially suppressed by JTT administration. Genes enriched in “Response to unfolded protein” also includes *Xbp1*, which encodes a transcription factor involved in the transcription of molecular chaperones (Lee et al., 2003). The genes enriched in “positive regulation of transcription from the RNA polymerase II promoter” (Supplementary Figure S3B), the most enriched GO term in the NYT reverse V-shaped recovery, included Arf4 (Smith and Mallucci, 2016), a transcription factor-related gene whose expression is upregulated by stress, and the inflammation-inducible transcription factors *Stat2* and *Stat3* (Pfitzner et al., 2004); although stress increases CREB activity and CREB target gene expression, *Creb1* (Boer et al., 2007; Manners et al., 2019) and CREB-related genes and (*Crebbp*, *Crem*) and CREB target genes (*Inhba*) (Walton et al., 2011), *Nr4a2* (Barneda-Zahonero et al., 2012) were also enriched.

Genes enriched in “Nervous system development” (Figure 3E) included *GSK3b*, which is reported to be involved in neuronal cell death and hippocampal atrophy (Sirerol-Piquer et al., 2011), and *Discam*, the overexpression of which inhibits dendrite branching (Alves-Sampaio et al., 2010; Sachse et al., 2019); in addition, *Sema4d* and *Efna5*, which have been reported to inhibit axon outgrowth as repulsive guidance factors (Wahl et al., 2000; Tasaka et al., 2012), were also enriched. Therefore, it is likely that stress occurs in the hippocampus of 19-week-old SAMP8 mice and that neurodevelopment-related genes expression id disrupted. However, Kampo formulas, particularly NYT, may prevent the onset of stress. To determine whether the major GO terms involved in this revere V-shaped recovery also exhibited differential expression between the two groups at the level of the overall gene sets, GSEA was performed (Supplementary Figures S6B-i–iv; Supplementary Table S8). Excluding “Nervous system development,” the other three GO terms “positive regulation of transcription from the RNA polymerase II promoter,” “Response to unfolded protein,” and “Protein folding,” exhibited a positive bias in P8CS compared with R1CS, whereas they were downregulated in P8NS. “Nervous system development” also exhibited a significant downregulated in P8NS. In P8JC, the negative bias was present but less pronounced than in the NYT group. These GSEA results are consistent with DEG-based GO analysis results and may represent a stress-adaptive response induced by Kampo administration.

### 3.4 The hippocampus of 19-week-old SAMP8 mice does not express PAS-positive granules but has a reduced number of cells and microglial gene expression

Microglial cell activation and DNA repair were ranked highest among the V-shaped recovery GO terms (Figure 3C, left). The heatmap of genes included in the terms (Figure 3D) showed that for “microglial cell activation,” the fractalkine (FKN, CX3CL1) receptor *Cx3cr1*, complement *C1q*, and Toll-like receptor (TLR) genes important for the innate immune response were expressed at low levels in SAMP8 mice and increased with Kampo administration. Although all of these factors may have detrimental effects on the CNS (Cho, 2019; Luo et al., 2019; Adhikarla et al., 2021), Kampo formulas may restore imbalances in microglial function in SAMP8 mice. *Naglu* encodes the lysosomal enzyme α-N-acetylglucosaminidase, which is required for heparan sulfate degradation (Prill et al., 2019) (Figure 3D). Neuropathological observations have revealed that SAMP8 mice begins to show PAS-positive granular structures, mainly in the hippocampus, at approximately 2∼3 months of age (Akiguchi et al., 2017). PAS staining allows the visualization of the accumulation of mucopolysaccharides such as heparan sulfate and glycogen. The accumulation of these mucopolysaccharides is caused by the failure of degrading enzymes in lysosomes. To explore the possibility that NYT inhibits mucopolysaccharide accumulation, PAS staining was performed using the mouse hippocampus, but few PAS-positive granules were observed in SAMP8 mice (Supplementary Figure S4B). There was also no change in hippocampal size among the groups (Supplementary Figure S4C). The length of the dentate gyrus (DG) (Supplementary Figure S4D), CA1 cell number (Supplementary Figure S4E), and CA3 cell number (Supplementary Figure S4F) were significantly lower in SAMP8 mice than in SAMR1 mice. Although administration of Kampo formulas did not lead to a significant recovery, these impairments were partially ameliorated by NYT.

Owing to the limited availability of hippocampal tissue samples, the prefrontal cortex (PFC), which is affected by neurological and psychiatric disorders together with the hippocampus (Ruggiero et al., 2021), was used to assess the synaptic protein content in SAMP8 mice. Postsynaptic density (PSD)-95 expression was slightly decreased in SAMP8 mice (p = 0.08) but was increased following NYT administration. However, there were no significant differences in the expression of the presynaptic marker synaptophysin (SYP) or the dendritic marker MAP2 (Figure 3F).

In summary, genetic changes are clearly evident from the earliest stages of hippocampal neurodegeneration, suggesting that during aging stress, NYT may protect the brain from neural damage by alleviating the dysregulation of the transcription of neurodevelopmental genes and stress response genes, as well as the decline in the expression of microglia-related genes.

### 3.5 NYT increases the expression of mitochondria-related genes

SAMP mice are highly sensitive to oxidative stress (Poon et al., 2004; Mori and Higuchi, 2019). Oxidative stress also causes of DNA damage, but many genes related to DNA repair were reduced in the P8CS group and exhibited a V-shaped recovery in the NYT group (Figure 3C, left, 3D). Relatedly, glutathione S-transferase (GST) (*Gsta4, Gsto1, Gstm4,* and *Gstm6*), the enzyme that catalyzes the addition of glutathione (GSH), which neutralizes reactive oxygen species, also showed a V-shaped recovery in the NYT group (Figure 3D, glutathione metabolic process).

Interestingly, in addition to V-shaped recovery genes, 1755 (Supplementary Figure S5A, green frame) unique mitochondria-related genes were enriched in the following GO terms, i.e., “mitochondrial respiratory chain complex I assembly,” “mitochondrial ATP synthesis coupled proton transport,” “aerobic respiration,” and “mitochondrial translation” (Supplementary Figure S5B, right). Mitochondrial dysfunction occurs with age, and patients with mitochondrial disease are also known to exhibit anxiety disorders (Filiou and Sandi, 2019). However, mitochondria-related genes were not differentially expressed between the R1CS and P8CS groups but were significantly increased in the P8NS group (Supplementary Figure S5C). The expression of *Atp5pb*, an ATP synthase, was increased in the JTT and NYT groups (Supplementary Figure S5C, mitochondrial ATP synthesis coupled protein transport). Although not associated with the top GO term, the gene expression levels of *Bcs1l*, a mitochondrial respiratory chain complex 3, and *Nlrx1*, a mitochondrially expressed receptor of the innate immune system, showed a V-shaped recovery, and the gene expression of *Chchd2*, which suppresses mitochondrial integration stress via elF2α (Ruan et al., 2022), was significantly higher in the JTT and NYT groups that in the P8CS group (Supplementary Figure S5D). Although indirectly, in PFA, the protein levels of NDUFB8, a subunit of mitochondrial complex I, also exhibited a tendency to increase in NYT, suggesting that these increase in mRNA levels may be reflected in protein levels (Supplementary Figure S5F).

Relatedly, “acetyl-CoA biosynthetic process” was the top enriched term for NYT-V recovery genes (Figure 3C, left,3D). Acetyl-CoA is known to decrease in the aging brain, and increased acetyl-CoA has been reported to have neuroprotective effects via histone acetylation (Currais et al., 2019). The inhibition of acetyl-CoA carboxylase 1 (ACC1), an enzyme involved in the conversion of acetyl-CoA to malonyl CoA, increases acetyl-CoA (Currais et al., 2019), and in this study, the expression level of the related gene, *Acaca*, markedly decreased with Kampo administration (Supplementary Figure S5E).

We also performed GSEA on the major GO terms involved in this V-shaped recovery (Supplementary Figure S6A; Supplementary Table S8). A comparison of R1CS and P8CS revealed that the enrichment score exhibited a negative bias in P8CS, whereas they were upregulated in P8NS. “DNA repair” was not affected by Kampo administration.

Regarding mitochondrial-related GO terms, GSEA revealed that “mitochondrial ATP synthesis coupled proton transport” (Supplementary Figure S5G-i; Supplementary Table S8) exhibited a significant upregulate under both NYT and JTT administration. Similarly, “mitochondrial translation” (Supplementary Figure S5G-ii) exhibited an upregulated in both administrations, with a more pronounced effect in the NYT group.

The concordance between DAVID enrichment analysis and GSEA suggests that the observed pathway alteration are driven both by subsets of genes with significant expression changes and by a systematic shift across the entire transcriptome induced by Kampo administration.

These findings suggest that, compared with JTT, NYT trends to provides greater protection to the hippocampus by improving mitochondrial function, energy metabolism, and neutralizing reactive oxygen species.

### 3.6 Effects of Kampo formulas on LPS-induced changes in gene expression

**FIGURE 4:**
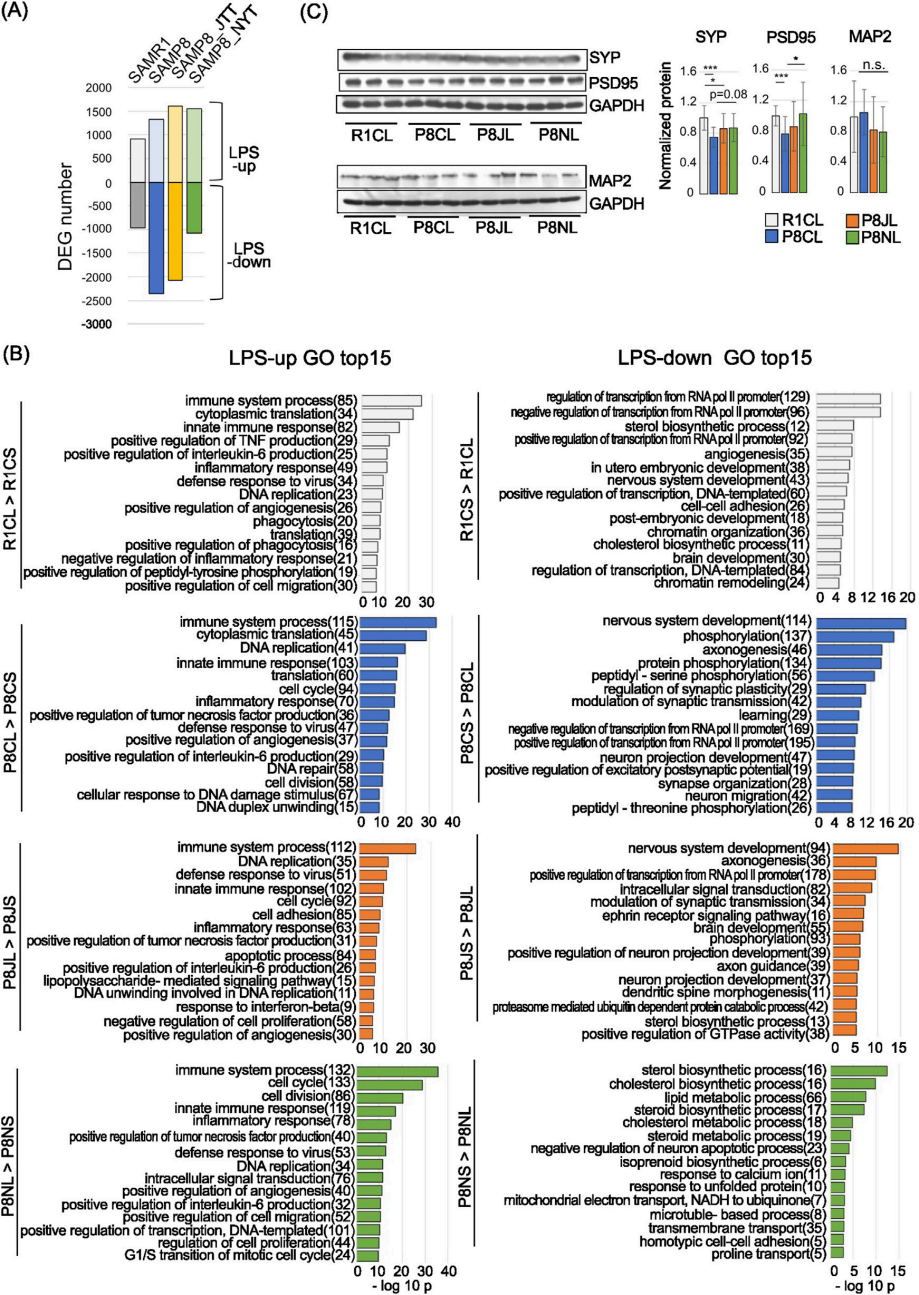
Changes in gene expression caused by LPS administration and GO analysis. **(A)** Number of genes whose expression levels were upregulated or downregulated after LPS administration. P < 0.05, using the likelihood ratio test. **(B)** GO analysis of genes whose expression levels were upregulated (left) or downregulated after LPS administration (right). Gene numbers are shown to the right of the term. Each of the top 50 GO terms belonging to the LPS-down category are listed in Supplementary Tables S1–S4. **(C)** Representative images of Western blot of MAP2, SYP and PSD95 in the PFC (left) and quantification of relative protein levels (right). GAPDH was used as a loading control. The data are shown as means ±SDs (n = 5). Experiments were independently repeated 2–3 times. n.s., no significant difference; *P < 0.05, ***p < 0.001 (Bonferroni correction). Uncropped images are shown in Supplementary Figure S8. Abbreviations: R1CS: SAMR1-control food-saline injection; P8CS: SAMP8-control food-saline injection; P8JS: SAMP8-JTT food-saline injection; P8NS: SAMP8-NYT food-saline injection; R1CL: SAMR1-control food- LPS injection; P8CL: SAMP8-control food-LPS injection; P8JL: SAMP8-JTT food- LPS injection; P8NL: SAMP8-NYT food-LPS injection.

In the hippocampus of SAMP8 mice at 19 weeks of age, there were no clear changes in genes associated with neuroinflammation at the genetic level. LPS exacerbates depressive behavior in mice 24 h after administration (Godbout et al., 2008; Henry et al., 2008; Zheng et al., 2021) because LPS activates macrophages and microglia, which release inflammatory mediators (Zheng et al., 2021; Fu et al., 2013). Thus, to investigate the efficacy of Kampo formulas in suppressing neuroinflammation, LPS was administered to 19 weeks old mice, and changes in hippocampal gene expression were examined 24 h later. The number of DEGs after LPS administration was lowest in SAMR1 mice and highest in SAMP8 LPS-down (2362, Figure 4A), suggesting that SAMP8 mice are more sensitive to LPS-induced effects. GO analysis confirmed the changes in DEGs caused by LPS in each group (Figure 4B). LPS induced immune, inflammatory response, and DNA replication-related genes in all groups (Figure 4B, left). Genes enriched in the “regulation of transcription from the RNA polymerase II promoter” and “nervous system development” were common among the SAMR1, SAMP8, and JTT groups, and genes enriched in “nervous system development,” “axonogenesis,” “regulation of synaptic plasticity,” and “leaning” were unique to the SAMP8 group (Figure 4B Right). Compared with that in SAMR1 mice, the nervous system in SAMP8 mice seems to be more vulnerable to LPS.

In the JTT group, as in the SAMP8 group, “nervous system development” was the top GO term; however, in the NYT group, there were very few nervous system-related GO terms, with genes mainly enriched in sterol biosynthetic process. The “sterol biosynthetic process” was not NYT specific, as it was also observed outside the top 15 GO terms in the other groups (Supplementary Tables S1–S4). Therefore, it is possible that the pre-administration of NYT may reduce susceptibility to LPS-induced nerve injury. Regarding synaptic markers in the PFC, SYP and PSD95 were significantly lower in the P8CL group than in the R1CL group, and tended to recover in the P8NL group (Figure 4C).

### 3.7 NYT alleviates LPS-induced nerve damage, and JTT alleviates LPS-induced inflammation

**FIGURE 5:**
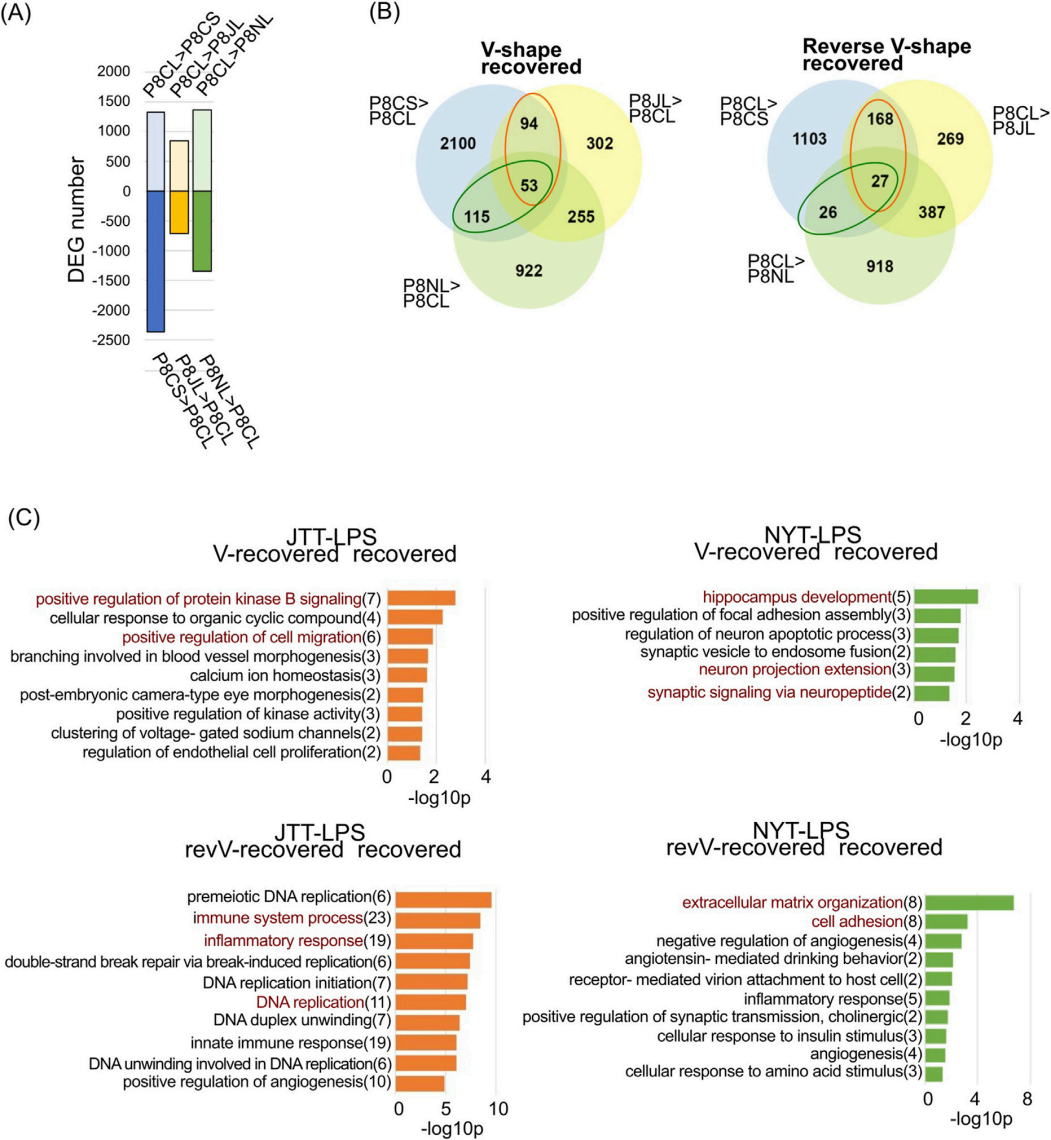
Changes in LPS-induced gene expression in SAMP8 mice pre-administrated with Kampo formulas. **(A)** Differential gene expression number between the P8CL group vs. the P8CS, P8JL, or P8NL group (p < 0.05, using the likelihood ratio test). **(B)** Venn diagram showing the number of genes differentially expressed between the P8CL group vs. the P8CS, P8JL, or P8NL group. The orange round frame indicates genes whose expression was restored by JTT, and the green round frame indicates genes whose expression was restored by NYT. **(C)** Gene ontology analysis of the biological processes of the JTT-recovered genes (left) and NYT-recovered genes (right). Gene numbers are shown to the right of the term.

**FIGURE 6:**
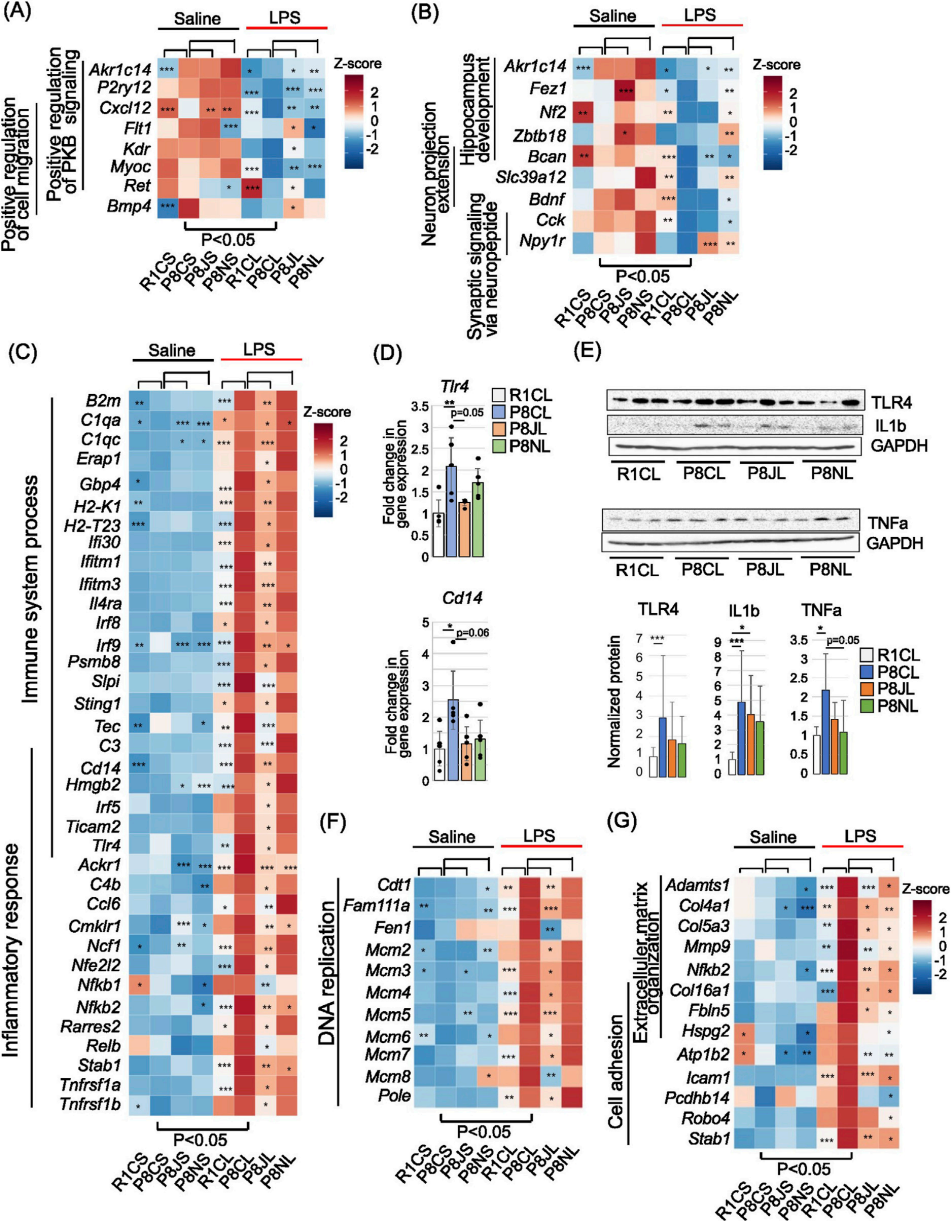
Overall gene levels belonging to a representative GO term for LPS-induced DEGs restored by prior administration of Kampo formulas. **(A)** Heatmap of the expression levels of genes associated with the GO terms “positive regulation of protein kinase B signaling” and “positive regulation of cell migration” in JTT-LPS V-shaped recovered genes shown in Figure 5C. **(B)** Heatmap of the expression levels of genes associated with the GO terms “hippocampus development,” “neuron projection extension” and “synaptic signaling via neuropeptide” in the NYT-LPS V-shaped recovered genes, as shown in Figure 5C. **(C)** Heatmap of the expression levels of genes associated with the GO terms “immune system process” and “inflammatory response” of the JTT-LPS reverse V-shaped recovered genes, as shown in Figure 5C. **(D)** *Tlr4* and *Cd14* mRNA expression levels determined via RT-qPCR are expressed as the fold change over the control group, shown as means ± SDs (n = 5 replicates). Individual data points are indicated by dots. *Gapdh* was used as an internal control. *p < 0.05 and **p < 0.01 (Bonferroni correction). **(E)** Representative images of Western blots of TLR4, TNFa and IL-1b in the PFC (upper) and quantification of relative protein levels (lower). GAPDH was used as a loading control. The data are shown as means ±SDs (n = 5). n.s., no significant difference; *p < 0.05 and ***p < 0.001 (Bonferroni correction). Uncropped images are shown in Supplementary Figure S8. **(F)** Heatmap of the expression levels of genes belonging to the GO term “DNA replication” of the JTT-LPS reverse V-shaped recovered genes in Figure 5C. **(G)** Heatmap of the expression levels of genes associated with the GO terms “extracellular matrix organization” and “cell adhesion” of the NYT-LPS reverse V-shaped recovered genes in Figure 5C. In **(A–C,F,G)**, these were significant differences between the P8CS group and the P8CL group, with p < 0.05. *P < 0.05, **p < 0.01, and ***p < 0.001: compared with P8CS in the saline group, and compared with P8CL in the LPS group (likelihood ratio test.).

To elucidate the mechanisms by which Kampo formulas suppress acute inflammation-induced neurodegeneration, LPS was administered to each group, and changes in gene expression were compared. In SAMP8 mice, after administration with LPS (P8CS vs. P8CL), these were 1324 upregulated genes and 2362 downregulated genes, with fewer variations between the P8CL group and the JTT group (Figure 5A), which is consistent with the proximity of P8CL and P8JL in the PCA (Supplementary Figure S2). The list of these DEGs is shown in Supplementary Table S7. In SAMP8 mice, we identified genes whose expression was altered by LPS but was rendered less affected by LPS following JTT or NYT administration (Figure 5B). Interestingly, despite the small number of DEG between the P8CL and P8JL groups, the number of genes with reverse V-shaped recovery with JTT was approximately 3.5-fold greater than that observed with NYT (Figure 5B, right). GO analysis of the genes recovered as a result of Kampo formulas revealed that those with V-shaped recovery in the JTT group were associated with terms related to cell migration (“positive regulation of protein kinase B signaling” and “positive regulation of cell migration”) (Figure 5C); those with V-shaped recovery in the NYT group were more associated with the development of the nervous system, for example, “hippocampus development,” “neuron projection extension,” and “synaptic signaling via neuropeptide.” For reverse V-shaped recovery genes in the JTT group, “immune system process,” “inflammatory response,” and “DNA replication-related factors” were the top-ranking terms. For reverse V-shaped recovery genes in the NYT group, “extracellular matrix organization” and “cell adhesion” were the most frequently observed terms. A heatmap of overall (Saline &LPS) gene expression levels is shown for the genes associated with these characteristic GO terms (Figure 6). The genes associated with “positive regulation of protein kinase B signaling” and “positive regulation of cell migration” among the GO term associated with V-shaped recovery genes in the JTT group exhibited overall decreased expression in all groups after LPS administration (Figure 6A). The P2Y receptor, *P2ry12* is a microglia signature gene (Yoo and Kwon, 2021), and consistent with a report that its expression decreases with a shift to an inflammatory phenotype (Gómez Morillas et al., 2021), *P2ry12* expression decreased after LPS administration. In particular, *P2ry12* expression was significantly lower in the P8CL group than in the other groups, suggesting that Kampo formulas may inhibit the shift to excessive inflammatory microglia. The expression of the chemokine *Cxcl12* is reportedly decreased in AD patients, as this decrease causes memory, and learning deficits and neurogenesis failure (Parachikova and Cotman, 2007; Abe et al., 2018). *Cxcl12* expression was significantly lower in SAMP8 group than in the other groups after both saline and LPS administration (Figure 6A). Myocilin mediates myelination in peripheral nerves (Kwon et al., 2013), and the LPS-induced reduction in this gene (*Myoc*) was suppressed in both Kampo formulas groups, indicating that Kampo formulas suppress the marked LPS-induced reduction in genes that act in neuroprotection.

Heatmaps of genes related to “hippocampus development,” “neuron projection extension,” and “synaptic signaling via neuropeptide,” which exhibited V-shaped recovery in the NYT group, revealed an overall decrease in expression upon LPS administraion; this decrease was suppressed in the R1CL and P8NL groups (Figure 6B). These genes include *Fez1*, which is involved in oligodendrocyte and myelin formation (Chen et al., 2017); *Nf2*, which is involved in axonal regeneration in peripheral neurogenic injury (Mindos et al., 2017); *Slc39s12*, which is required for neurite outgrowth (Davis et al., 2021); brain-derived neurotrophic factor (BDNF), which is important for neural activity and synaptic plasticity (Oh et al., 2019; Wang et al., 2019), and the cholecystokinin gene (*Cck*), which has neuroprotective effects in Alzheimer’s disease and Parkinson’s disease (Reich and Holscher, 2024). These findings suggest that compared with JTT, NYT may more effectively mitigate LPS-induced neuronal damage.

The expression levels of genes related to the “immune system process” and “inflammatory response,” which exhibited reversed V-shaped recovery in the JTT group, were increased by LPS in all groups (Figure 6C). Among the LPS-administrated groups, the increase was significantly lower in the R1CL and P8JL groups than in the P8CL group, and the expression of both TLR4, an LPS receptor expressed in microglia, and the coreceptor Cd14 were suppressed in the JTT group. In addition, the interferon-regulatory factor (IRF) family (*Irf5, Irf8,* and *Irf9*) is associated with microglial activation, and the TLR4-mediated activation of the NF-κβ transcription factors (*Nfkb1* and *Nfkb2*), TNFα receptors (*Tnfrsf1a* and *Tnfrsf1b*) (Lawrence, 2009; Ji et al., 2022) and complements (*C1qa, C1qc, C3,* and *C4b*) was also suppressed in the JTT group.

RT-qPCR data also revealed significant increases in hippocampal *Tlr4* and *Cdc14* in the P8CL group, with suppressed expression observed in the JTT group (Figure 6D). Protein quantification in the PFC revealed no clear suppression of TLR4 or the release of the microglia-released cytokines TNFα and IL-1β in the JTT group. There was no increase in these factors in NYT group, but rather, these was a trend toward the suppressing of TNFα (p = 0.05) (Figure 6E).

The DNA replication-related genes that showed reverse V-shaped recovery in the JTT group were mostly DNA helicases (*Mcm2-8*) (Figure 6F). *Cdt1*, which is important for cell cycle progression, is expressed at low levels in the G0 phase, increases in the G1 phase and is also essential for loading MCM into DNA (Pozo and Cook, 2016). Fam111a is involved in promoting DNA replication, but excess Fam111a increases DNA damage and apoptosis (Rios-Szwed et al., 2023). Although cell division is generally suppressed in neurons, stress can activate cell cycle reentry in neurons, resulting in cell cycle arrest, an increase in neurons with duplicated chromosomes, and other forms of genomic instability (replication stress) that can lead to cell death (Yurov et al., 2011). The genes included in “extracellular matrix organization” and “cell adhesion,” which showed reverse V-shaped recovery in the NYT group, were also suppressed in the JTT group (Figure 6G). Moreover, these genes are related to leukocyte infiltration into the vascular endothelium, and include collagen (*Col4a1, Col5a3,* and *Col16a1*), *Mmp9*, and *Icam1*.

These findings suggest that in LPS-induced inflammation, JTT may be involved in mitigating inflammatory responses via the TLR4/NF-κβ pathway and vascular permeability, and mitigating neural damage via the suppression of DNA replication stress, whereas NYT may act to maintain genes involved in axonal regeneration and neuroprotection and alleviate inflammatory responses through the suppression of vascular permeability.

### 3.8 Effects of Kampo formulas on microglia-related genes

**FIGURE 7:**
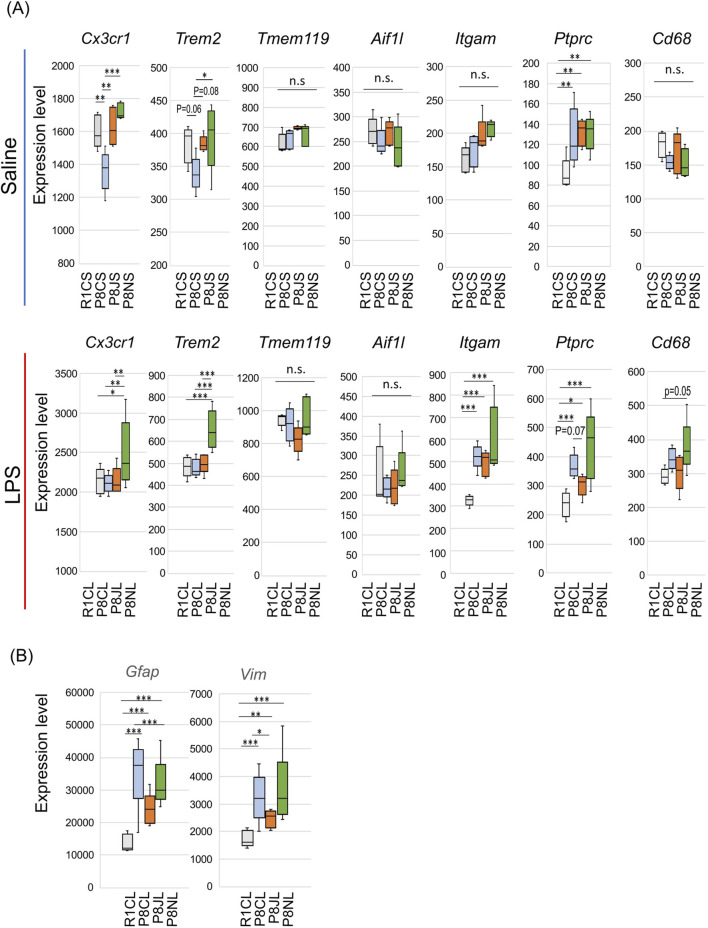
Differences in the effects of JTT and NYT on glia. **(A)** Gene expression levels of microglia-related genes. Upper: saline-administrated group, lower: LPS-administrated group. *P < 0.05, **P < 0.01, and ***P < 0.001 (n = 5) vs. the P8CS group or the P8CL group, using the likelihood ratio test. **(B)** Gene expression levels of active astrocyte-related genes in LPS-administrated group. *P < 0.05, **P < 0.01, and ***P < 0.001 (n = 5) vs. the P8CL group, using the likelihood ratio test.

On the basis of our experimental results and the possibility that both Kampo formulas affect microglia-related gene expression, we focused on microglial marker genes. In the saline-administrated group, the expression of the FKN receptor *Cx3cr1* in SAMP8 mice was significantly reduced, and in the JTT and NYT groups, *Cx3cr1* expression were restored (Figures 3D, 7A upper). The other marker, triggering receptor expressed on myeloid cells 2 (*Trem2*) was also slightly lower in SAMP8 mice and significantly greater in the NYT group. Although JTT increases the expression of the activation marker CD11b and Iba1 in microglia and promotes phagocytosis (Liu et al., 2008; Liu et al., 2014), our data revealed no difference in the expression levels of *Aif1*(Iba1) or *Itgm* (CD11b) between the Kampo-administrated groups and the SAMP8 group. Moreover, no difference was observed in the expression of the microglia signature genes *Tmem119* or *CD69*, which are involved in the phagocytosis of microglia (Figure 7A, upper). However, *Ptprc*, the gene encoding CD45, which is highly expressed in bone marrow-derived macrophages and other immune cells, was expressed at significantly lower in the R1CS group than in all the other groups, suggesting that early aging stress in SAMP8 promotes the induction of peripheral monocytes in the brain (Figure 7A upper).

Microglia remove excess synapses via the complement system, and in the saline group, C1q (*C1qa, b,* and *c*) expression was significantly higher in the Kampo formulas groups compared than in the P8CS group. However, no differences were observed for complement *C3* and *C4a* (Supplementary Figure S7 upper), suggesting that the classical complement pathway activity and the activation of C3-mediated microglial phagocytosis do not occur in the hippocampus of SAMP8 mice at 19 weeks of early aging.

Interestingly, the administration of LPS resulted in the significant upregulation of *Cx3cr1* and *Trem2* expression in microglia in the NYT group (Figure 7A lower). *Ptprc* and *Cd68* expression also tended to be higher in the P8NL group than in the other groups. Inflammatory cytokines from microglia induce reactive astrocyte and neuronal cell death (Liddelow et al., 2017), and the levels of the reactive astrocyte markers *Gfap* and vimentin (*Vim*) (Hol and Pekny, 2015) were significantly increased in the P8CL and P8NL groups and suppressed in the JTT group (Figure 7B). The complements all showed a pattern of more pronounced increases in expression in P8CL and P8NL groups, indicating that the classical complement pathway was activated by LPS (Supplementary Figure S7 lower). CX3CR1 is expressed only in microglia in the brain, and CX3CR1 gene knockdown exacerbates the release of proinflammatory factors by LPS and exacerbates depressive-like behavior due to the persistent activity of microglia in the PFC and hippocampus (Luo et al., 2019). In cultured cells, CX3CR1 decreases LPS-induced release of inflammatory factors from microglia (Mattison et al., 2013). Mutations in TREM2 increase the risk of late-onset AD and frontotemporal dementia (Borroni et al., 2014), and microglia deficient in TREM2 have a shortened migration distance to nerve damage and a reduced ability to respond to nerve damage (Mazaheri et al., 2017). Conversely, the overexpression of TREM2 has been shown to alleviate spatial cognitive deficits and suppresses inflammatory cytokine production in a mouse model of AD (Ruganzu et al., 2021), suggesting that TREM2 activation is protective in neurodegenerative diseases such as AD.

Taken together, these data suggest that microglia in the hippocampus of 19-week-old SAMP8 mice have a reduced ability to protect against microglial neuronal damage and that NYT may protect nerves against acute inflammatory stress by increasing the expression of the Cx3cr1 and Trem2 genes. JTT also promoted the recovery of the *Cx3cr1* gene but inhibited the excessive inflammatory response to acute inflammatory stress mainly through the suppression of the TLR4 pathway and astrocyte activity.

## 4 Discussion

Greater severity of depressive symptoms is associated with memory decline, and clinical depression in older adults is recognized as a risk factor for the development of dementia (Lu et al., 2021; Yin et al., 2024). In this study, we used 4-month-old (19-week-old) SAMP8 mice, which exhibit accelerated aging along with anxiety- and depression-like behavioral phenotypes, to compare the effects of JTT and NYT on frailty. Furthermore, SAMP8 mice subjected to LPS-induced acute inflammation were employed as a model of exacerbated neuroinflammatory depression, in order to further evaluate the effects of these Kampo formulas. JTT and NYT inhibited weight loss in SAMP8 mice, a finding that is consistent with the effects of these Kampo formulas in the treatment of anorexia. Although food intake was not directly measured, anorexia associated with aging may have contributed to the observed weight loss in SAMP8 mice. Additionally, in behavioral tests, consistent with previous reports, SAMP8 mice presented emotional disturbances such as anxiety, apathy, and increased mobility (Yanai and Endo, 2016; Pacesova et al., 2022), as well as a decrease in the number of neurons in the hippocampus (Pacesova et al., 2022). JTT and NYT prevented the worsening of apathy, and the decrease in neuron number was alleviated by NYT. On the basis of gene expression levels in the hippocampus, multiple sites and pathways were candidates for the mechanisms of action: 1) suppression of stress-inducible transcription factors and genes related to nervous system development, 2) increased mitochondrial gene expression and alleviation of oxidative stress, 3) maintenance of microglial function, and 4) the microglial TLR4-mediated inflammatory response.

### 4.1 Suppression of the stress response by Kampo formulas

In the hippocampus of 4-month-old SAMP8 mice, the expression of various stress-responsive transcription factors, i.e., *Atf4* (Hetz and Mollereau, 2014; Smith and Mallucci, 2016), which is transcribed via the stress-induced activation of the transcription initiation factor eIF2α, the stress-induced transcription factor CREB and its target factors, the inflammation-induced transcription factor STAT-family, and the unfolded protein response-activated transcription factor Xbp1 (Hetz and Mollereau, 2014), is upregulated. The unfolded protein response also activates Atf4, whose target genes include those involved in protein folding (Hetz and Mollereau, 2014; Smith and Mallucci, 2016). That finding is consistent with the increase in protein folding-related genes in SAMP8 mice (Figure 3E). Although it has been reported that the loss of CREB downregulates the expression of inflammation- and immune-related genes (Manners et al., 2019), it is also possible that the stress is not sufficient to increase the expression of stress-responsive transcription factors, especially with NYT administration. The GO term “Nervous system development” included several genes that are increased in inflammatory and neurodegenerative diseases, such as Gsk3b, the expression of which was downregulated by Kampo formulas, suggesting that NYT and JTT, to some extent, reduce the susceptibility of the hippocampus to stress. Similarly, we speculate that administration of Kampo does not result in an increase in stress-induced unfolded proteins (Figure 3E). Furthermore, especially in the NYT group, the expression level of DNA repair-related genes was restored, suggesting that the cells were less susceptible to damage (Figure 3D). However, despite the apparent restoration of gene expression, the hippocampus of 4-month-old SAMP8 mice did not clearly show PAS-positive granular structures, as previously reported (Akiguchi et al., 2017), the presence of which occurs before extensive brain deposition associated with neurodegenerative diseases. This finding suggests that Kampo formulas exert a regulatory effect on variations in gene expression levels, even prior to overt pathological degenerative changes. Intron retention is a stress response mechanism that maintains cellular homeostasis, and abnormal intron accumulation or a decrease in intron retention has been observed in aging and neurodegenerative diseases. Japanese Kampo formulas such as JTT have been shown to restore intron retention, which fluctuates with age (Okada et al., 2021; Vu et al., 2022; Okada et al., 2024). It is plausible that the genes whose expression levels were restored in this study may be associated with the restoration of intron retention. Further analyses are necessary to better understand the molecular mechanisms of Kampo formulas.

### 4.2 Mitochondria and Kampo formulas

Dysfunctional mitochondria accumulate with age, and the increased mitochondrial production of reactive oxygen species (ROS) accelerates cellular aging. The results of basic studies using cultured cells have indicated that NYT alleviates fatigue by increasing the amount and activity of mitochondria (Takeda et al., 2018). Consistent with these finding, our data revealed that NYT markedly increased the expression of many mitochondrial membrane respiratory chain NADH dehydrogenase (Complex I) genes and mitochondrial ribosomal genes while increasing the expression of oxidative stress detoxification-related genes (GSTs), indicating an antioxidant effect (Figure 3D). Acetyl-CoA is an important metabolite that is produced within mitochondria and utilized for energy production by the tricarboxylic acid (TCA) cycle. In accordance with the findings of decreased acetyl-CoA levels in the aging brain (Currais et al., 2019), acetyl-CoA synthesis-related genes were decreased in SAMP8 mice, and Kampo formulas significantly increased these levels (Figure 3D). Currais et al. (2019) reported that AD drug candidate compounds increase acetyl-CoA in aging SAMP8 mice by inhibiting ACC1, an enzyme involved in acetyl-CoA catabolism, and have neuroprotective effects through histone acetylation, enhancing memory and maintaining mitochondrial homeostasis. These findings reveal a parallel relationship between the effects of JTT and NYT on acetyl-CoA and mitochondria, similar to those of AD drug candidate compounds, and support the hypothesis that these Kampo formulas may resist neurotoxicity.

Mitochondria are energy providers that facilitate local neuronal translation, and neuronal protein synthesis in dendrites also occurs in mitochondria (Rangaraju et al., 2019; Liang, 2021). Indeed, as shown in Supplementary Figure S5B, the top GO terms for the Kampo formulas groups and P8CS group were “Cytoplasmic translation” and “Translation,” and most of the genes were ribosomal protein large (Rpl) and small (Rps) subunit mRNAs and translation initiation factors, such as eIF4E (Supplementary Table S5). In the NYT group, genes enriched in “Translation” also exhibited V-shaped recovery (Figure 3C). Several ribosomal proteins decrease with age in neurodegenerative diseases, reducing protein synthesis (Evans et al., 2019; Koren et al., 2019) and are also involved in promoting axonal regeneration and repair (Schaeffer and Belin, 2022; Xing et al., 2023). Although many mitochondrial and ribosomal protein genes were not significantly lower in SAMP8 mice than in SAMR1 mice in this study, Kampo formulas may be able to promote the onset of axonal regeneration through an increase in these genes to compensate for the loss of function rather than expression.

### 4.3 Effects of Kampo formulas on microglia

Microglia play multiple physiological roles in the central nervous system, including synaptogenesis, synaptic pruning, maintenance of neurons such as neurite outgrowth, the promotion of myelination, and phagocytosis. Microglia are also activated in inflammatory conditions such as infection and stress, during which they produce inflammatory mediators, and excessive inflammatory cytokine production has been associated with depressive disorders (Yirmiya et al., 2015). LPS is used to induce acute inflammatory stress to generate mouse models of depression, in which LPS activates microglia via the TLR4/NF-κB pathway. NYT weakens LPS-induced microglial activity (Alim et al., 2022). NYT and JTT both contain Ginseng Radix, and ginsenosides derives from Ginseng Radix inhibit LPS-induced inflammatory cytokines and upregulate the expression of anti-inflammatory cytokines (Paik et al., 2019). JTT has LPS-like immunostimulatory effects, increases the number of CD11b-positive microglia in the brain, and inhibits Aβ deposition (Hara et al., 2010); however, the detailed regulatory mechanisms remain unclear. Our results regarding the expression levels of microglia-related genes revealed that during aging stress (in the saline group), Kampo formulas restored *Cx3cr1* and *Trem2* expression, which was reduced in SAMP8 mice. FKN/CX3CR1 are involved in a complex network of neurons and glia. CX3CR1 deletion causes cognitive and synaptic deficits (Wang et al., 2022), impairs neuron-microglia reactivity to chronic stress (Milior et al., 2016), and decreases hippocampal neurogenesis (Bachstetter et al., 2011). TREM2 expression is downregulated during aging (Butovsky and Weiner, 2018), and microglia lacking TREM2 have a reduced ability to respond to nerve damage (Mazaheri et al., 2017). Other microglial markers were less affected, indicating that NYT and JTT may specifically restore genes that protect neurons. Furthermore, only complement C1q was increased in the NYT and JTT groups, suggesting that it functions differently from C3 and C4a. Although C1q levels usually increase dramatically with aging and neurodegenerative diseases (Cho, 2019), why C1q levels particularly increased with Kampo is unclear. Microglia are a major source of C1q in the brain (Fonseca et al., 2017) and have protective functions in the brain, including proper neuronal synaptic pruning in the healthy brain. Recently, a role for the complement-independent phagocytosis of C1q was reported (Espericueta et al., 2020). Thus, NYT and JTT may restore microglia that become less stress-responsive with age, thereby protecting the nervous system.

In acute inflammatory stress induced by LPS, the neuroprotective effect of NYT appeared to be ineffective because compared with SAMR1 mice, both SAMP8 mice and NYT-administrated mice presented excessive increases in immune and inflammatory response-related genes, astrocyte activity and complement activity. In contrast to expectations, however, there was no significant increase in IL1b or TNFα; rather, there was a tendency for TNFα to be decreased in the PFC. The hippocampus also expresses *P2ry12*, which is reduced in inflammatory microglia, and *Bdnf*, a neurotrophic factor released by microglia, suggesting that NYT maintains neuroprotective microglia.

*Cx3cr1* and *Trem2* were significantly increased only in the NYT group. Interestingly, TREM2 has neuroprotective effects in cerebrovascular disease by suppressing TLR4 expression and the downstream NF-κB pathway and suppressing the expression of inflammation-induced cytokines (Liu et al., 2022; Awuah et al., 2025). In the NYT group, not only was the expression of *Trem2* upregulated but the expression of *Tlr4* was also high. Since the data were obtained 24 h after LPS administration, it is quite possible that LPS-induced proinflammatory microglia switch to an anti-inflammatory phenotype by overexpressing TREM2 after this time point. Despite the proposed mechanisms through which FKN/CX3CR1 protects against neurotoxicity (Luo et al., 2019), the molecular mechanisms by which increased *Cx3cr1* expression inhibits microglial activation and inflammatory cytokine release remain to be elucidated. As previously mentioned, CX3CR1 deficiency has adverse neurological effects; however, others have reported that chronically stressed animals exhibit increases in CX3CR1 and inflammation-inducing factors in the brain (Rossetti et al., 2016). We cannot rule out the possibility that this increase in the NYT group represents an adverse effect. However, further studies are necessary to ascertain whether this occurrence is transient or if it gives rise to chronic inflammatory stress.

Unlike NYT, JTT suppressed the expression of the LPS receptor *Trl4*, the coreceptor *Cd14*, and the downstream NF-κB gene; furthermore, it suppressed excessive increases in immune- and inflammation-related genes, astrocyte activity, and complement activity-related genes. To our knowledge, few reports demonstrated that JTT is as effective as NYT in alleviating psychiatric symptoms such as anxiety and depression. However, JTT may appropriately regulate TLR4 signaling induced by LPS, as well as downstream microglial morphological changes and cytokine production, suggesting its potential to prevent LPS-induced neuronal damage.

## 5 Limitations and future perspectives

The present study focused on the early stage of aging, specifically using 4-month-old SAMP8 mice that exhibited depression-like behavior. Therefore, it remains unclear whether the beneficial effects of these Kampo formulas also extend to more advanced stages of aging or to conditions in which depression-like behavior and learning and memory function have further deteriorated after LPS administration. To ensure experimental stability and reproducibility, only male mice were used in this study. However, since late-life depression is more prevalent in women, future investigations should also include female mice to better capture the clinical features of geriatric depression and to explore potential sex-specific differences in the effects of Kampo formulas.

Moreover, it should be noted that LPS serves as a model of acute, TLR4-dependent inflammation, which does not necessarily reflect the chronic neurodegenerative processes associated with aging. Future studies should therefore aim to examine the relationship between acute inflammation models and the long-term changes that occur with natural aging in wild-type mice. Studies using aged wild-type mice will be essential to verify whether the beneficial effects of Kampo formulas observed here can be generalized to age-related neurodegeneration.

In addition, due to the limited availability of hippocampal samples, we were unable to directly verify protein-level recovery in the hippocampus, restricting our findings to indirect evidence obtained from the PFC. We acknowledge that the sample size in the present study was minimal, and recognize that larger sample sizes would further strengthen the reliability of the findings. Therefore, future studies should employ larger sample size and larger cohorts of animals to allow more thorough and definitive validation of hippocampal gene and protein expression, while also incorporating analyses at the level of specific cell subtypes and pharmacochemical assays in serum. Such approaches will be essential for providing a more comprehensive and mechanistic understanding of the effects of Kampo formulas through long-term and multifaceted investigations.

## 6 Conclusion

In conclusion, this study provides novel insights into the mechanisms of two Kampo formulas in alleviating depression-like behavior associated with early aging, based on comparative analysis of gene expression profiles. Both NYT and JTT alleviated depression-like behaviors associated with aging and suppressed the increase in stress-responsive transcription factors. Notably, NYT further promoted the expression of mitochondrial and DNA repair genes, thereby reducing oxidative stress and contributing to neuroprotection. Under acute inflammatory stress, NYT preserved or enhanced the expression of neuroprotective microglial genes such as *Cx3cr1*, *Trem2*, and *Bdnf*, restoring stress resistance. In contrast, JTT attenuated excessive inflammation and immune responses by suppressing microglial TLR4 signaling and astrocytic activation. Taken together, while JTT exhibits acute anti-inflammatory effects through inhibition of TLR4-mediated signaling, NYT appeared to promote adaptive stress responses by maintaining the neuroprotective functions of microglia. Nevertheless, as these findings are based on transcriptomic changes in the early stages of hippocampal aging, further studies are warranted to elucidate the detailed molecular mechanisms underlying these effects.

## Data Availability

The data presented in the study are deposited in the NCBI BioProject repository, accession number PRJDB20202. The DRA accession numbers are available at https://www.ncbi.nlm.nih.gov/Traces/study/?acc=DRP014360. Further inquiries can be directed to the corresponding authors.
