# Supplementary figures and images for: Distinct neuroprotective and anti-inflammatory effects of Kampo formulas ninjinyoeito and juzentaihoto in depression-like SAMP8 mice
Source: Front Pharmacol. 2025 Oct 24;16:1600176. doi: 10.3389/fphar.2025.1600176 (PMC12592136; doi:10.3389/fphar.2025.1600176)

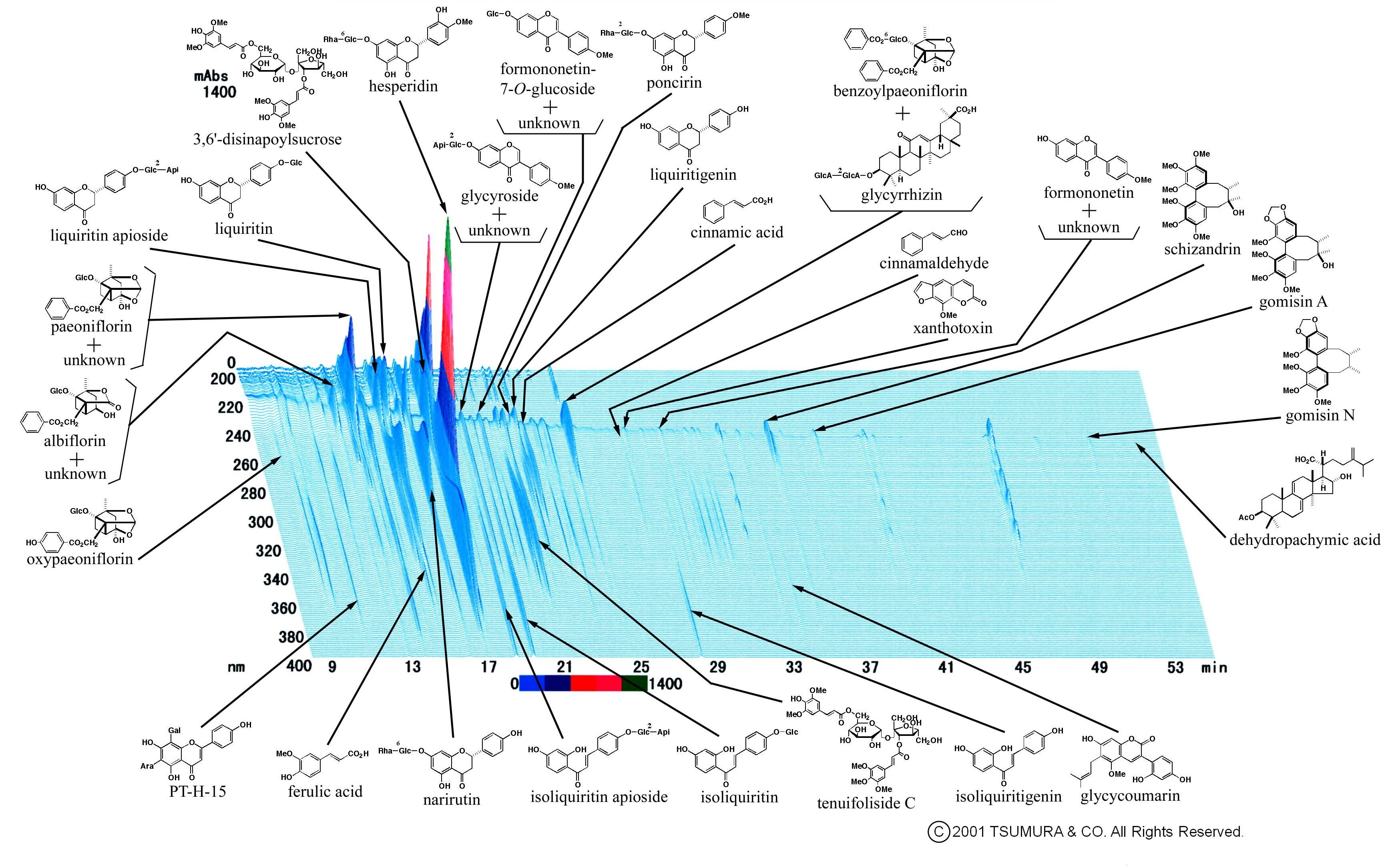

Supplement: Supplementary file 3 [file Image1.jpeg]

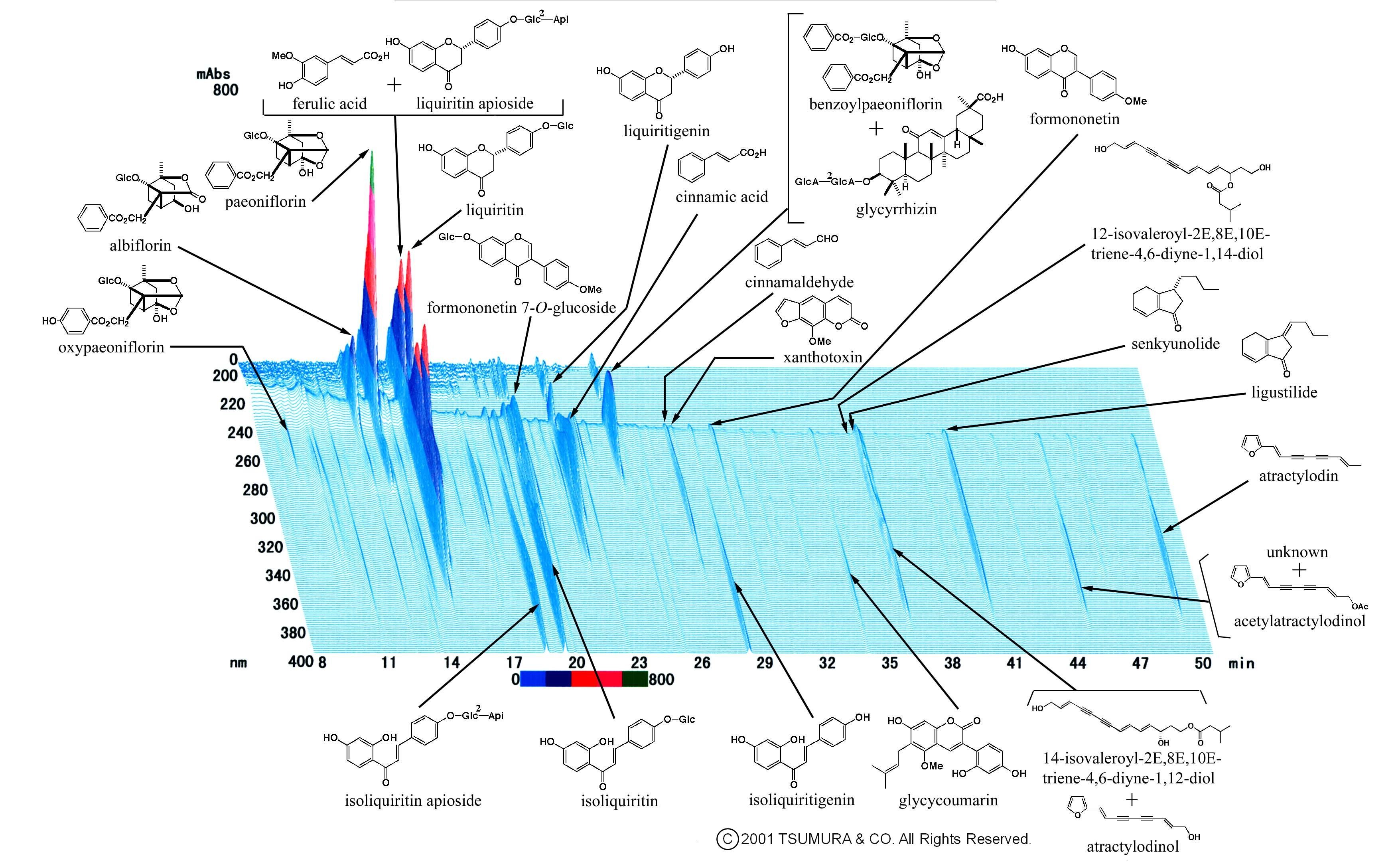

Supplement: Supplementary file 5 [file Image2.jpeg]
