# Supplementary material for: Distinct neuroprotective and anti-inflammatory effects of Kampo formulas ninjinyoeito and juzentaihoto in depression-like SAMP8 mice
Source: Front Pharmacol. 2025 Oct 24;16:1600176. doi: 10.3389/fphar.2025.1600176 (PMC12592136; doi:10.3389/fphar.2025.1600176)
Supplement: Supplementary file 4 [file Supplementaryfile1.pdf]

Supplementary Materials

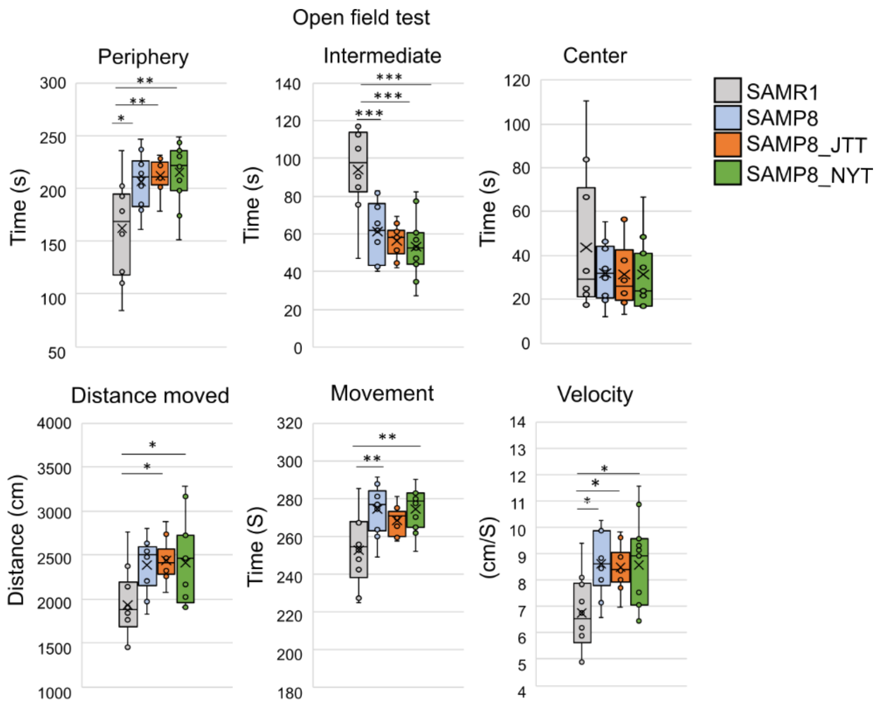

Supplementary Figure 1. Open field test Box plot showing median, quartiles, and bars indicating the maximum and minimum values. Crosses represent the means. *p<0.05, **p<0.01, ***p<0.001 (Tukey HSD). SAMR1, SAMP8, SAMP8_JTT: n=10, SAMP8_NYT: n=11

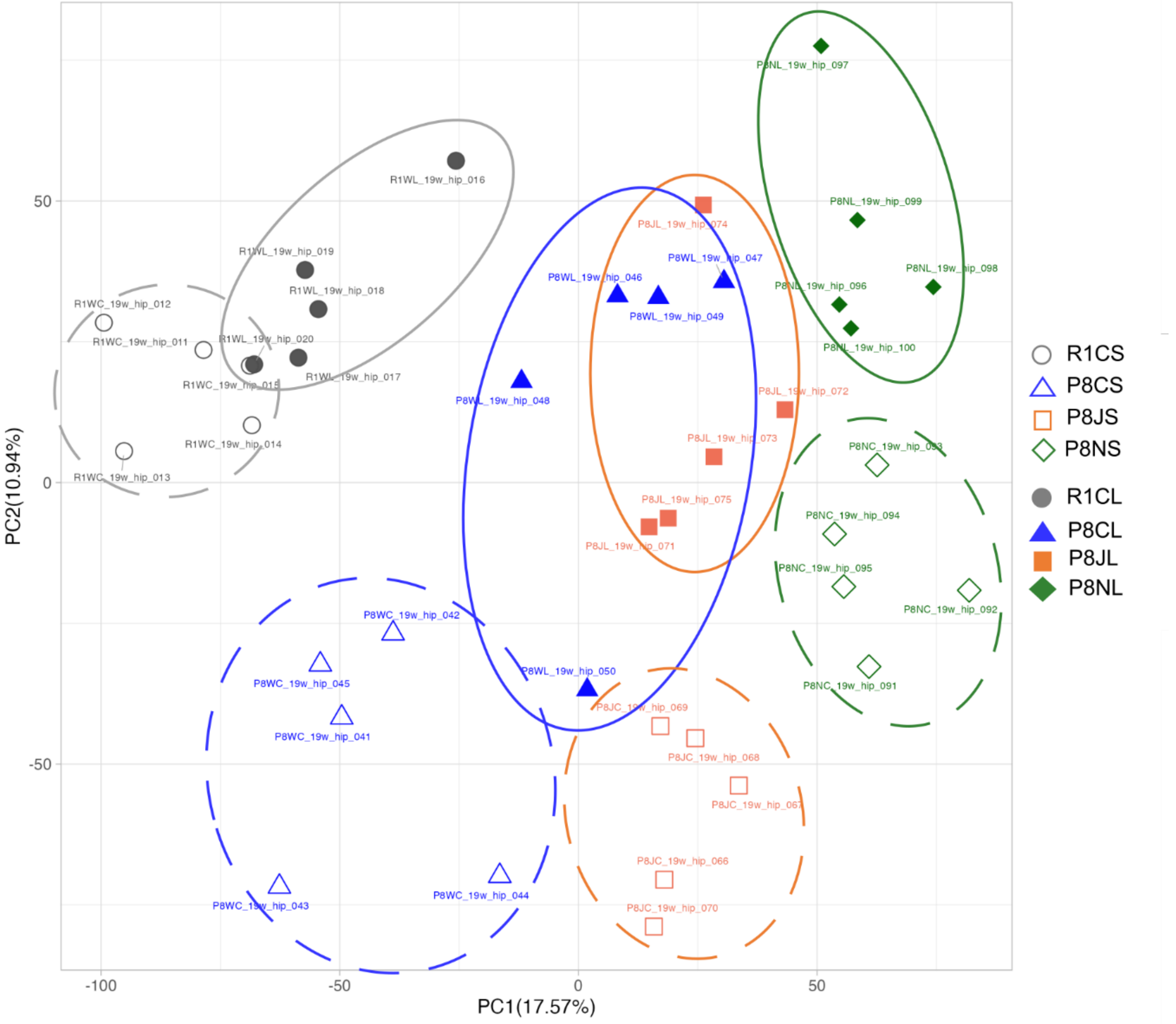

Supplementary Figure 2. Principal Component Analysis(PCA) of RNA-seq gene expression data

PCA analysis showed each group clusters. The dashed line indicates the saline administration group. The solid line indicates the LPS-administered group.

Abbreviations: R1CS: SAMR1-control food- saline injection; P8CS: SAMP8-control food- saline injection; P8JS: SAMP8-JTT food- saline injection; P8NS: SAMP8-NYT food- saline injection; R1CL: SAMR1-control food- LPS injection; P8CL: SAMP8-control food- LPS injection; P8JL: SAMP8-JTT food- LPS injection; P8NL: SAMP8-NYT food- LPS injection

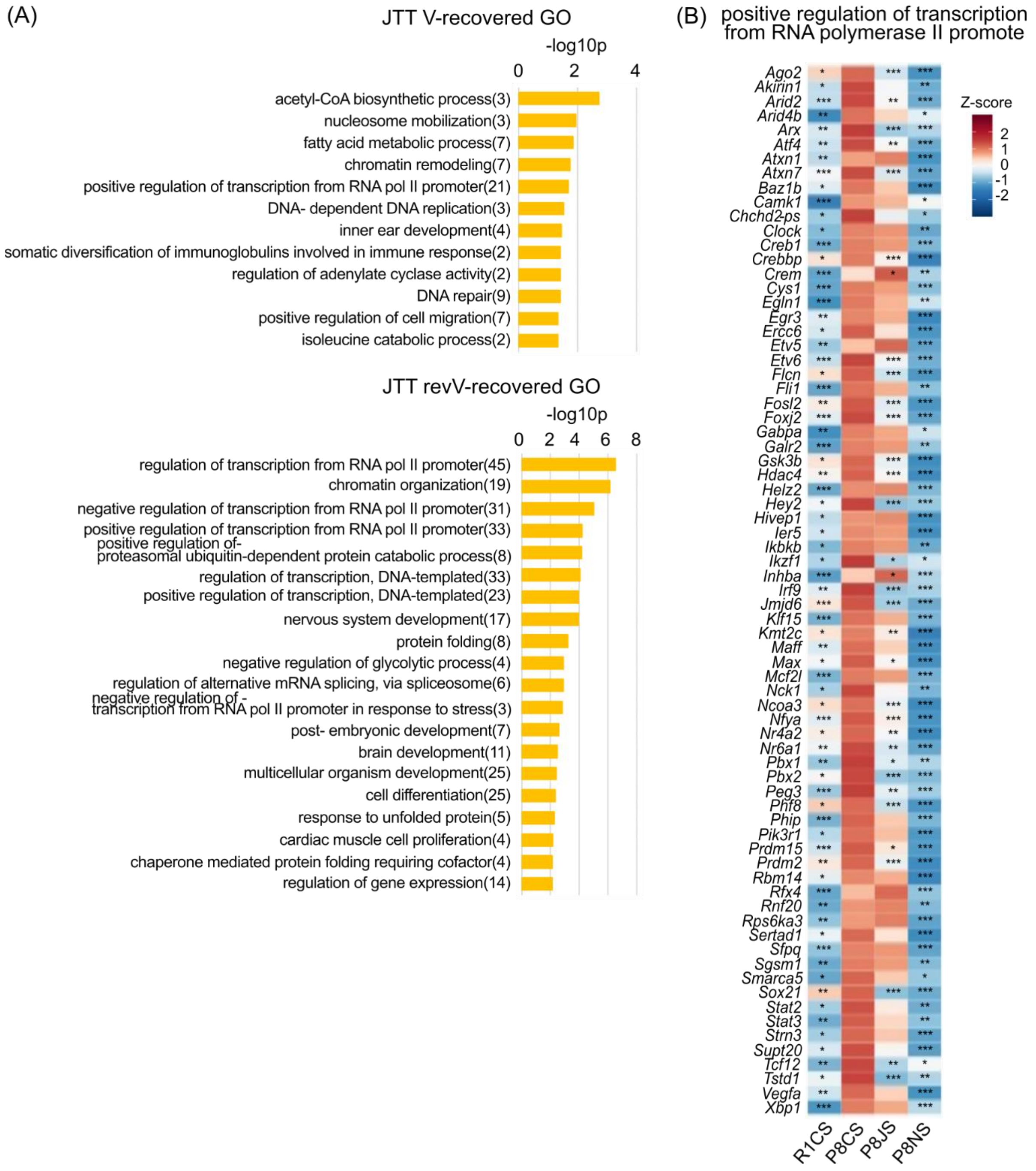

Supplementary Figure 3. NYT downregulated transcription related genes expression

(A) Gene ontology (GO) analysis of biological processes of JTT-V shaped recovered genes (upper) and JTT- reverse V shaped recovered genes (lower) from Figure 3B. Gene numbers are shown to the right of the term. (B) Heat map of differentially expressed genes that included in the GO terms of "positive regulation of transcription from RNA polymerase 2 promoter" in the NYT-reverse V recovered genes in Figure 3C. *P < 0.05, **P < 0.01, and ***P < 0.001 (n =5) vs. P8CS, using the likelihood ratio test.

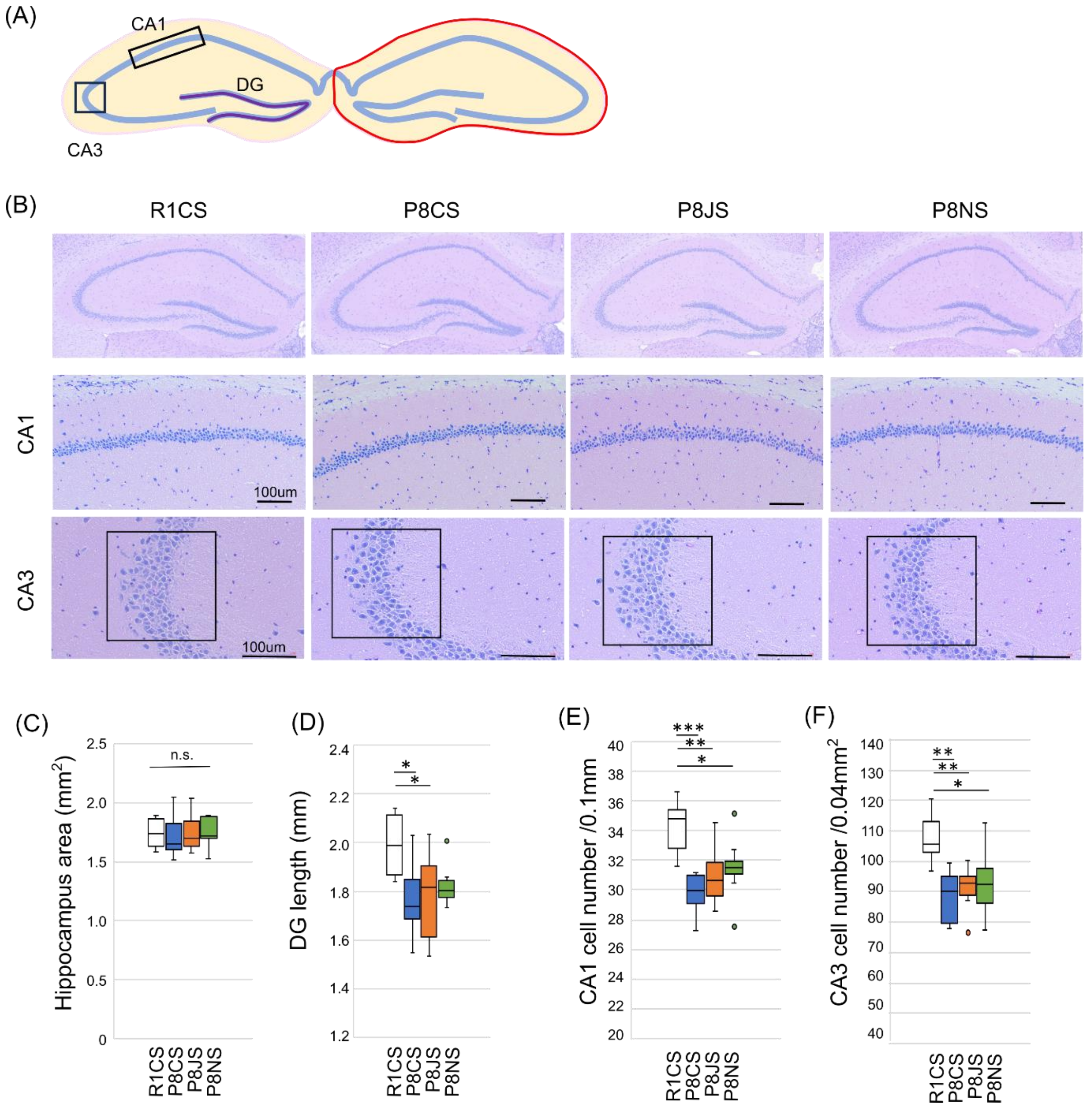

Supplementary Figure 4. Analysis of hippocampus area and cell number.

(A) Schematic representation of the region analyzed. (B) Representative captures of hippocampus regions in R1CS, P8CS, P8JS, and P8NS. Coronal sections of the hippocampus were PAS-stained and HE-stained. Images were obtained with a x4 (upper images), x10 (middle CA1 images), x20 (bottom CA3 images), with a Nikon microscope. The black frame in CA3 is the area where cell counts were measured (0.04 mm^2). Scale bar, 100 µm. (C) Hippocampus area. (D) DG (dentate gyrus) length. (E) Mean CA1 number of cells per 0.1 mm in 0.6 mm. (F) Mean CA3 number of cells per 0.04 mm^2. All graphs are mean ± SD and analyzed by TukeyHSD test; *P < 0.05, **P < 0.01, and ***P < 0.001 (N=4, counting left-right brains per individual).

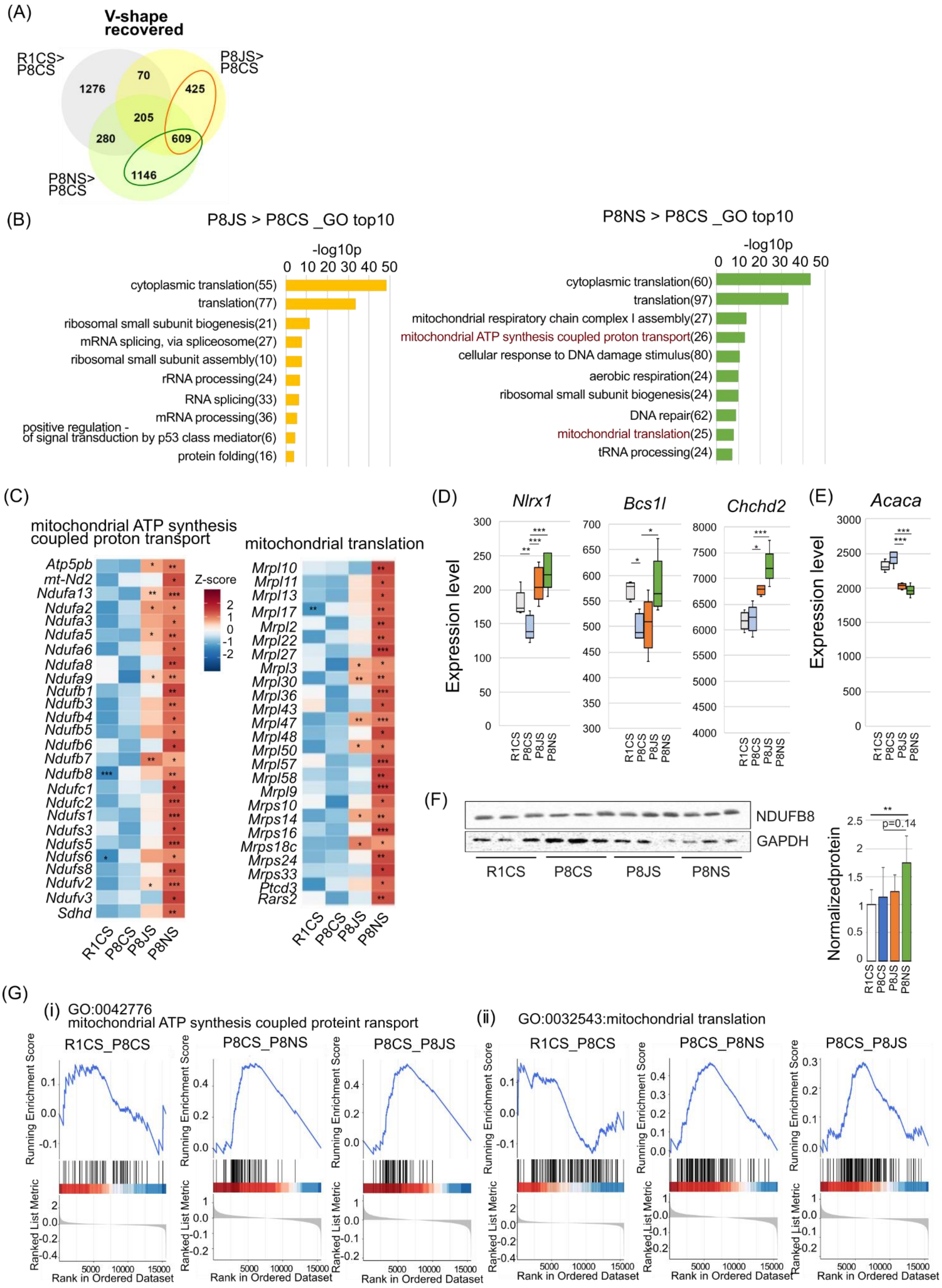

(A)
V-shape recovered
R1CS> P8CS
P8JS> P8CS
P8NS> P8CS
1276
70
425
205
280
609
1146
(B)
P8JS > P8CS _GO top10
-log10p
0 10 20 30 40 50
cytoplasmic translation(55)
translation(77)
ribosomal small subunit biogenesis(21)
mRNA splicing, via spliceosome(27)
ribosomal small subunit assembly(10)
rRNA processing(24)
RNA splicing(33)
mRNA processing(36)
positive regulation - of signal transduction by p53 class mediator(6)
protein folding(16)
P8NS > P8CS _GO top10
-log10p
0 10 20 30 40 50
cytoplasmic translation(60)
translation(97)
mitochondrial respiratory chain complex I assembly(27)
mitochondrial ATP synthesis coupled proton transport(26)
cellular response to DNA damage stimulus(80)
aerobic respiration(24)
ribosomal small subunit biogenesis(24)
DNA repair(62)
mitochondrial translation(25)
tRNA processing(24)
(C)
mitochondrial ATP synthesis coupled proton transport
Atp5pb
mt-Nd2
Ndufa13
Ndufa2
Ndufa3
Ndufa5
Ndufa6
Ndufa8
Ndufa9
Ndufb1
Ndufb3
Ndufb4
Ndufb5
Ndufb6
Ndufb7
Ndufb8
Ndufc1
Ndufc2
Ndufs1
Ndufs3
Ndufs5
Ndufs6
Ndufs8
Ndufv2
Ndufv3
Sdhd
R1CS P8CS P8JS P8NS
Z-score
2 1 0 -1 -2
mitochondrial translation
Mrpl10
Mrpl11
Mrpl13
Mrpl17
Mrpl2
Mrpl22
Mrpl27
Mrpl3
Mrpl30
Mrpl36
Mrpl43
Mrpl47
Mrpl48
Mrpl50
Mrpl57
Mrpl58
Mrpl9
Mrps10
Mrps14
Mrps16
Mrps18c
Mrps24
Mrps33
Ptcd3
Rars2
R1CS P8CS P8JS P8NS
(D)
Nlrx1
Bcs1l
Chchd2
Expression level
R1CS P8CS P8JS P8NS
(E)
Acaca
Expression level
R1CS P8CS P8JS P8NS
(F)
NDUFB8
GAPDH
R1CS
P8CS
P8JS
P8NS
Normalizedprotein
p=0.14
R1CS P8CS P8JS P8NS
(G)
(i) GO:0042776
mitochondrial ATP synthesis coupled proteint ransport
R1CS_P8CS
P8CS_P8NS
P8CS_P8JS
(ii) GO:0032543:mitochondrial translation
R1CS_P8CS
P8CS_P8NS
P8CS_P8JS
Running Enrichment Score
Ranked List Metric
Rank in Ordered Dataset

Supplementary Figure 5. NYT upregulated mitochondria related genes expression
(A) Venn diagram showing the genes differentially expressed among P8CS vs R1CS, P8JS, or P8NS (same as Figure 3B left). Numbers in circles indicate the number of genes with statistically significant changes in expression ($P < 0.05$) vs. P8CS, using the likelihood ratio test. The orange round frame indicates genes that were upregulated in P8JS compared to P8CS, except for V-shaped recovered genes. The green round frame indicates genes that were upregulated in P8NS compared to P8CS, except for V-shaped recovered genes. (B) Gene ontology (GO) analysis of biological processes of P8JC>P8CS genes (left) and P8NC>P8CS genes (right). Gene numbers are shown to the right of the term. Genes belonging to these GO terms are listed in **Supplementary Table 5**. (C) Heat map of differentially expressed genes that included in the GO terms in P8NS > P8CS of "mitochondrial ATP synthesis coupled proton transport" and "mitochondrial translation" in the P8NC>P8CS genes. (D) Gene expression levels of mitochondria-related genes *Nlrx1*, *Bcs1l* and *Chch2d.* (E) Gene expression levels of acetyl-CoA carboxylase apha genes *Acaca*. In (C), (D) and (E): *$P < 0.05$, **$P < 0.01$, and ***$P < 0.001$ ($n = 5$) vs. P8CS, using the likelihood ratio test. (F) Representative images of Western blots of NDUFB8 in the prefrontal cortex; the bar graph shows the quantification of relative protein levels. GAPDH was used as a loading control. The data are shown as means ± SDs (n=5). Experiments were independently repeated 2–3 times. **$p < 0.01$ (Bonferroni correction). Uncropped images are shown in **Supplementary Figure 8**. (G) Gene Set Enrichment Analysis (GSEA) plot showing the enrichment of (i) "mitochondrial ATP synthesis coupled proton transport" and (ii) "mitochondrial translation" in R1CS versus P8CS (left), P8CS versus P8NS (center), and P8CS versus P8JS (right). The y-axis represents the enrichment score, and the x-axis indicates the ranked list of genes. The vertical lines indicate the positions of genes belonging to the gene set, and the running enrichment score (blue line) reflects the degree of enrichment at each position. The peak of the curve corresponds to the maximum enrichment score, indicating the most significantly enriched gene set.

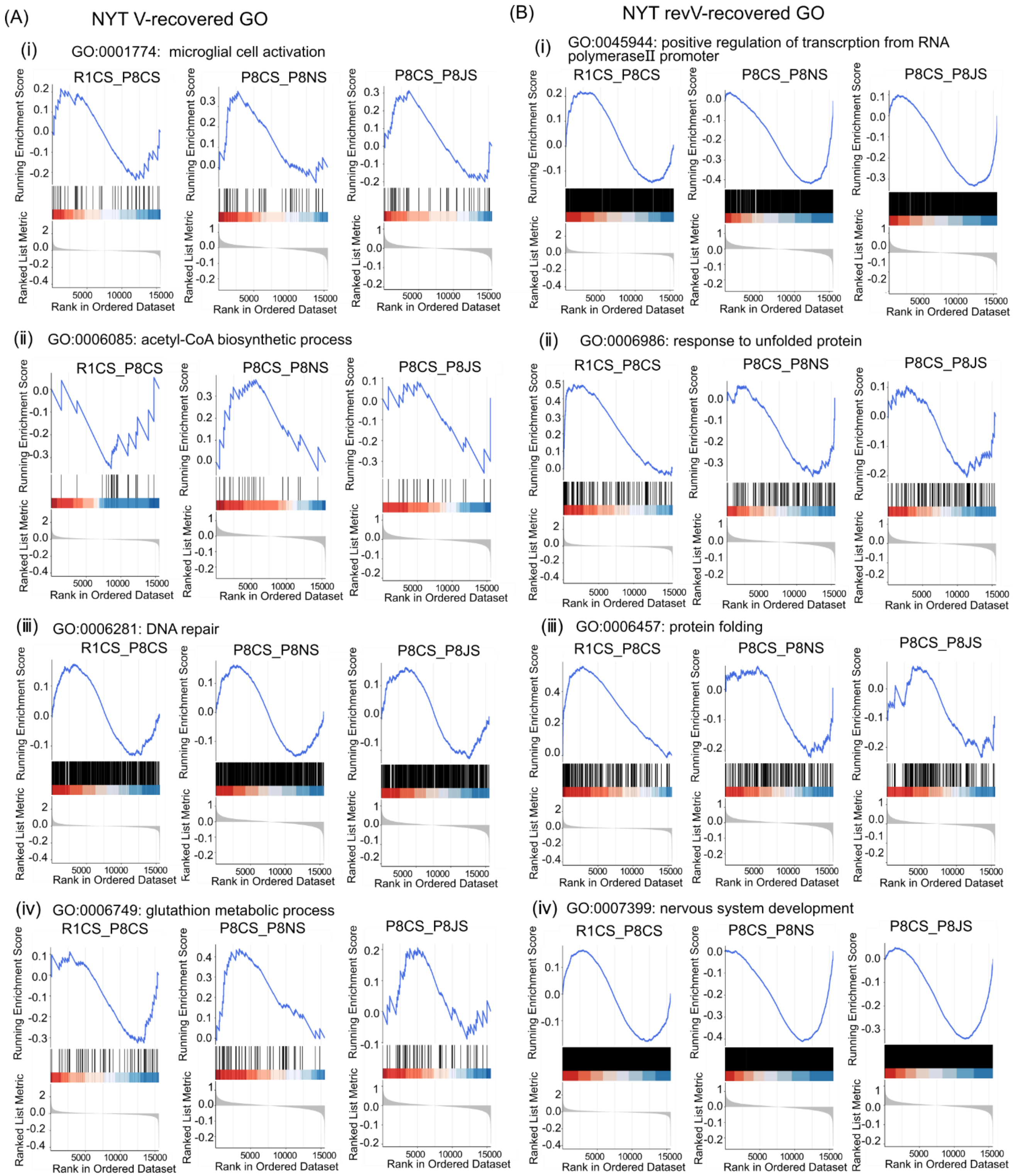
(A)
NYT V-recovered GO
(i) GO:0001774: microglial cell activation
(ii) GO:0006085: acetyl-CoA biosynthetic process
(iii) GO:0006281: DNA repair
(iv) GO:0006749: glutathion metabolic process
(B)
NYT revV-recovered GO
(i) GO:0045944: positive regulation of transcrption from RNA polymeraseII promoter
(ii) GO:0006986: response to unfolded protein
(iii) GO:0006457: protein folding
(iv) GO:0007399: nervous system development
R1CS_P8CS
P8CS_P8NS
P8CS_P8JS
Running Enrichment Score
Ranked List Metric
Rank in Ordered Dataset

Supplementary Figure 6. The GSEA plots of the key GO terms are highlighted in red in Figure 3C.

(A) GSEA plots for NYT V-recovered Gene Ontology (GO) terms: (i) "microglial cell activation," (ii) "acetyl-CoA biosynthetic process," (iii) "DNA repair," and (iv) "glutathione metabolic process," shown for R1CS vs. P8CS (left) and P8CS vs. P8NS (right). (B) GSEA plots for NYT reverse V-recovered GO terms: (i) “positive regulation of transcription from RNA polymerase II promoter”, (ii) “response to unfolded protein”, (iii) “protein folding”, and (iv) “nervous system development”, shown for R1CS vs. P8CS (left), P8CS versus P8NS (center), and P8CS versus P8JS (right). The y-axis represents the enrichment score, and the x-axis indicates the ranked list of genes. The vertical lines indicate the positions of genes belonging to the gene set, and the running enrichment score (blue line) reflects the degree of enrichment at each position. The peak of the curve corresponds to the maximum enrichment score, indicating the most significantly enriched gene set.

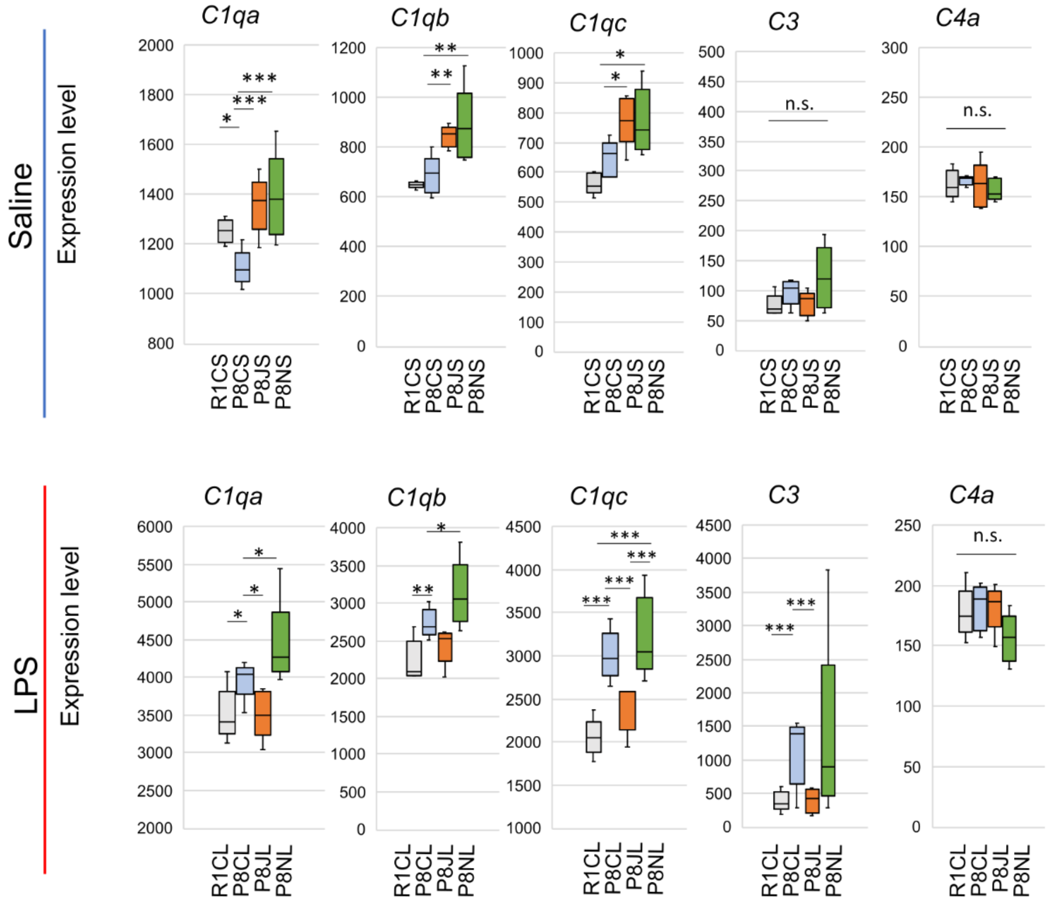

Supplementary Figure 7. Differential effects of JTT and NYT on gene expression levels in complements.

Gene expression levels of complement genes. Upper: Saline-treated group, Lower: LPS-treated group. *P < 0.05, **P < 0.01, and ***P < 0.001 (n =5) vs. P8CS or P8CL, using the likelihood ratio test.

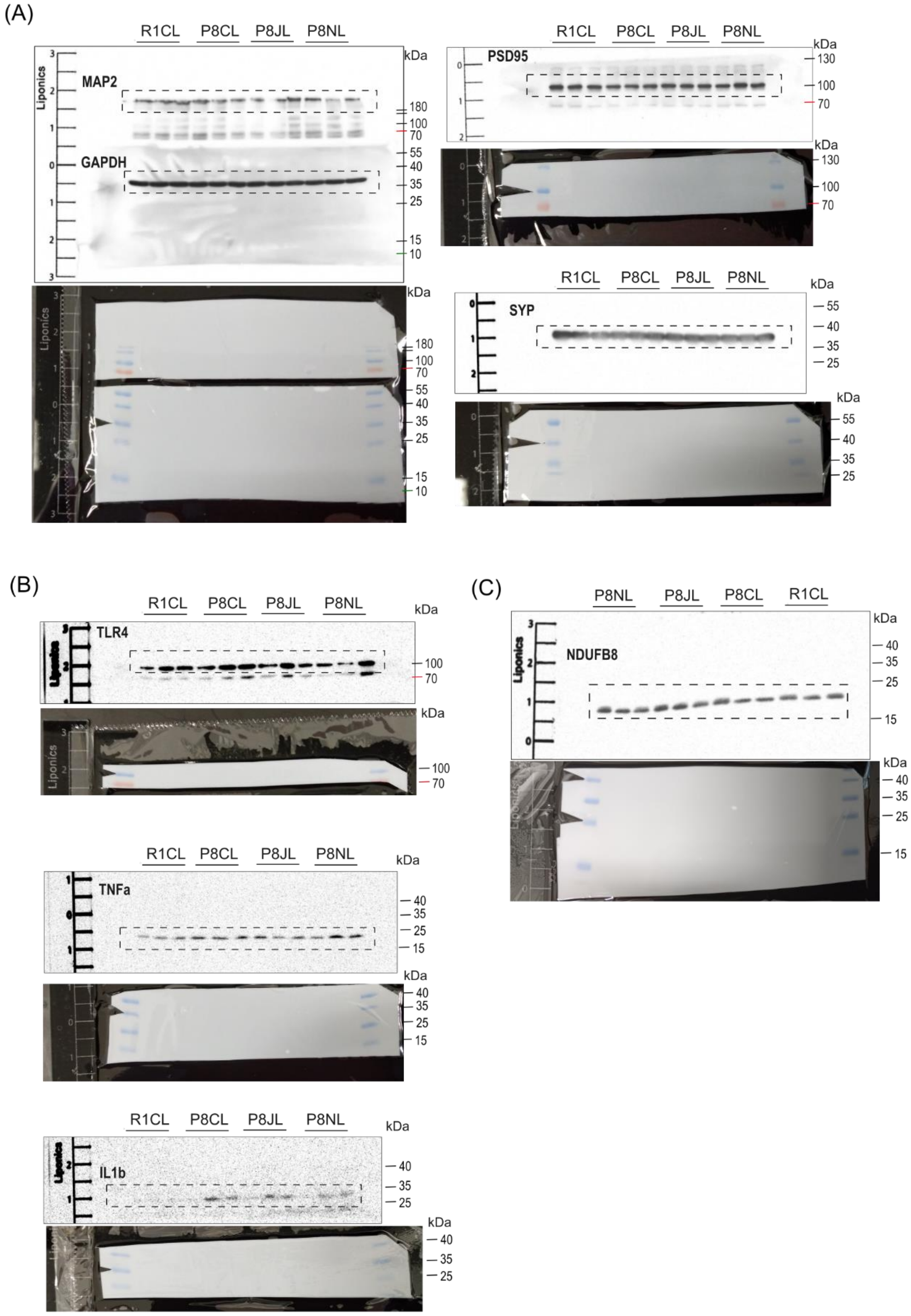
(A)
R1CL
P8CL
P8JL
P8NL
MAP2
GAPDH
Liponics
kDa
180
100
70
55
40
35
25
15
10
PSD95
130
SYP
(B)
TLR4
TNFa
IL1b
(C)
NDUFB8

Supplementary Figure 8. Uncropped Western blot images.

Uncropped Western blot images with the approximate regions used for figure preparation are shown. For reference, both band exposure images and membrane images are provided to verify the molecular weight markers. Membranes were often cut to enable blotting for multiple antibodies. (A) corresponds to Figure 4C, (B) corresponds to Figure 6E, and (C) corresponds to **Supplementary Figure 5F**.
